# Effectiveness of text messaging interventions on prevention, detection, treatment, and knowledge outcomes for sexually transmitted infections (STIs)/HIV: a systematic review and meta-analysis

**DOI:** 10.1186/s13643-018-0921-4

**Published:** 2019-01-08

**Authors:** Darlene Taylor, Carole Lunny, Petra Lolić, Orion Warje, Jasmina Geldman, Tom Wong, Mark Gilbert, Richard Lester, Gina Ogilvie

**Affiliations:** 1 University of British Columbia, 1147 Research Road, Rm ARTS 154, Kelowna, BC V1V 1V7 Canada; 2 University of British Columbia, 2329 West Mall, Vancouver, BC V6T 1Z4 Canada; 3 BC Centre for Disease Control, 655 W12th Ave, Vancouver, BC Canada; 4 Vancouver Island Health Authority, 1952 Bay Street, Victoria, BC V8R 1J8 Canada; 5 Health Canada/Santé Canada, 200 Eglantine Driveway, Room 1913A, Ottawa, ON K1A 0K9 Canada; 6 University of British Columbia, Research Pavilion, Rm 566, 828 W 10th, Vancouver, BC V5Z 1 M9 Canada; 7 University of British Columbia, Box 42, 4500 Oak Street, Vancouver, BC V6H 3N1 Canada

**Keywords:** Text messaging, Sexually transmitted diseases, Delivery of health care, Evidence-based medicine, Meta-analysis

## Abstract

**Background:**

Rates of STIs continue to rise worldwide, and novel evidence-based interventions such as text messaging aimed at improving client services are needed. We conducted a meta-analysis to evaluate text messaging to support STI/HIV prevention and treatment interventions.

**Methods:**

We included articles that reported findings from randomized controlled trials (RTCs) involving adults and youth who were at risk of acquiring (or who currently had) a STI and/or HIV, a text message and comparator intervention, and reported provided outcome data on adherence to STI/HIV treatments. Articles were excluded if they were not published in English. We only included studies that have full-text publications so certainty and risk of bias assessments could be performed. Eight databases were searched to retrieve articles published between 1996 and March 2017. The Cochrane risk of bias tool was used and certainty of the evidence was assessed using GRADE. Effect estimates were pooled using a random effects model.

**Results:**

A total of 35 RCTs were found, 6 of which were considered at low risk of bias. Eight studies found an increased association using text messaging in appointments attended compared to standard care (OR 1.64, 95% CI 1.28 to 2.10). Participants receiving text messages had an increase in HIV testing compared to standard care (*n* = 6; OR 1.73, 95% CI 1.39 to 2.15). Ten text messaging RCTs measuring adherence using micro-electro-mechanical systems (MEMS) pill counts has a non-significant association (OR 1.17, 95% CI 0.95–1.45) while five studies measuring adherence by self-report was found to be significant (OR 1.64, 95% CI 1.28–2.11).

**Conclusions:**

The effectiveness of text message interventions is equivocal. While text messaging has the potential to enhance the delivery of STI/HIV interventions, program planners are encouraged to evaluate any SMS intervention to ensure it is achieving the desired result.

**Systematic review registration:**

PROSPERO CRD42013006503

**Electronic supplementary material:**

The online version of this article (10.1186/s13643-018-0921-4) contains supplementary material, which is available to authorized users.

## Background

Increasing rates of sexually transmitted infections (STIs) remain a major public health challenge worldwide, posing a challenge to its control and appropriate management. According to the World Health Organization (WHO), “more than 1 million STIs are acquired every day worldwide” with a yearly incidence rate of 357 million per year [1]. In many western jurisdictions including Canada, Australia, and the USA, reported rates of genital chlamydia and gonorrhea are increasing. In Canada, for example, rates of genital chlamydia have steadily increased from 133/100,000 population in 1999 to 304/100,000 in 2015 [2]. Similarly, the rate of gonorrhea infections increased from 20/100,000 reported cases in 1999 to 50/100,000 reported cases in 2015 [2]. The latest data from Australia shows that gonorrhea has increased by 63% over the past 5 years (between 2012 and 2016; 62 to 101 per 100,000) [3]. These increased rates heighten concerns of sequelae such as pelvic inflammatory disease (PID), infertility, and increased vulnerability to acquiring HIV. Furthermore, rates of reported cases of infectious syphilis, continue to rapidly increase among men who have sex with men (MSM) in Canada, the USA, and Western Europe [4–6].

Surveillance, prevention, and control of chlamydia, gonorrhea, syphilis, herpes, human papillomavirus (HPV), and human immunodeficiency virus (HIV) infections consume a substantial direct cost [7]. In contrast, text messaging interventions may be cost-effective and can potentially reach a large number of people across large jurisdictions [8, 9]. Evidence-based prevention and control interventions are key to controlling these communicable diseases [10].

Cell phones are widely used globally with an estimated 62.9% of the population worldwide owning a mobile phone. This estimate is expected to exceed five billion by 2019 [11]. As such, the popularity of this technology, including the capability to transmit text messages (also referred to as short message service [SMS]), may provide an opportunity to reach populations. SMS has become the most common mode of communication among almost five billion mobile phone users worldwide [12] and provides an easy way for health staff to administer an intervention. Text messages have been used to remind patients about clinic appointments, to notify patients that it is time for STI re-testing, and to facilitate patient communication with their health professionals related to their sexual health. Text messaging can be delivered manually or through automated systems [13] and can act as an intervention via two-way communication, which can allow for interactive support by health care providers, or via one-way program-initiated communication to deliver key health messages to the user [14–16].

A large body of literature has emerged demonstrating the effectiveness of SMS on STI/HIV control [17–20]. However, individual study results are markedly disparate. This points to the need for a comprehensive systematic review and meta-analysis. Several systematic reviews have been published examining SMS interventions in relation to broad questions such as attendance in clinics [21], sexual health in adolescents [22–24], reminder interventions to retest [25], and countless others. Three previous meta-analyses [26–28] and one network meta-analysis [29] have been conducted that pooled the results of randomized controlled trials (RCTs) on the effectiveness of text messaging interventions on HIV adherence and viral load. These programs take on varied purposes for engaging individuals through the prevention-to-treatment spectrum; however, the evidence of the effectiveness of text messaging interventions must be clear before such programs could be transitioned to policy and recommended as a tool for STI prevention and control globally.

We are improving previous research by including and reviewing more current studies, including only RCTs, assessing the risk of bias of the included RCTs, and grading the overall certainty of evidence [30]. We conducted a systematic review and meta-analysis of RCTs aimed at evaluating the impact of SMS on STI/HIV prevention, detection, and control.

## Methods

This systematic review was registered with PROSPERO (CRD42013006503), the protocol has been published [31], and a PRISMA statement checklist has been completed (Additional file 1).

### Search strategy

This systematic review and meta-analysis was performed according to the Preferred Reporting Items for Systematic review and Meta-Analysis (PRISMA) guidelines [32]. The search was conducted in two phases in order to provide an up-to-date review. The initial search (phase 1) was conducted in the Cochrane Database of Systematic Reviews, MEDLINE, ACP Journal Club, Database of Abstracts of Reviews, EMBASE, EBM Reviews, and included articles published from 1996 to August 31, 2013. We chose to limit the search starting at 1996 as it was thought that this was when SMS first became widely used. In addition, the table of contents for the following journals was reviewed: *Sexually Transmitted Diseases*, *Sexually Transmitted Infections*, and *AIDS Patient Care and STDs*. Grey literature was searched using Google and Grey Literature Report (www.greylit.org) to identify studies that may have been missed in the abovementioned searches. Medical subject headings (MeSH) and keywords included mobile health, mHealth, cell phone, mobile phone short message services, text messaging, texting, SMS, MMS, communication technologies, patient monitoring devices, wireless technologies, STI testing, sexually transmitted diseases, sexually transmitted infections, HIV, chlamydia, gonorrhea, herpes, *Trichomonas vaginalis*, and syphilis. The search strategies are included in Additional file 2. The second search (phase 2) was conducted in MEDLINE and mhealthevidence.org (Additional file 3) using the same search terms. Limiting our search to MEDLINE and mhealthevidence.org was done in this phase due to a lack of resources. This included articles published between September 1, 2013, and March 30, 2017. We also examined the reference lists of any relevant systematic reviews retrieved.

### Eligibility criteria

RCTs examining the effect of SMS on STI/HIV prevention and control outcomes among adults and youth published in English between January 1, 1996, and March 30, 2017, were included. For phase 2, we only included studies that were identified in MEDLINE and mhealthevidence.org between 2013 and 2017. For the purposes of this review, SMS was defined as a text message that is delivered to a mobile phone either manually or by an automated system. We included only RCTs published in English as we did not have the financial and human resources to translate non-English studies. A detailed description of the inclusion/exclusion criteria using a PICO (Population, Intervention, Comparison, Outcome) format can be found in Additional file 4.

### Selection of studies

Titles and abstracts were independently examined for eligibility by two reviewers (PL, OW). Disagreements were resolved by a third reviewer (DT). Studies that were deemed eligible based on title and abstract were then reviewed by two independent researchers to confirm eligibility. Forward searching was done using the included references. Our updated search involved screening by one reviewer (CL) with a second reviewer checking the full text of included studies (DT). No test of agreement between reviewers was conducted for either of the search phases. All discrepancies were discussed until consensus was achieved.

### Data extraction

Data were extracted independently by two reviewers (PL, OW) using a standardized pre-tested data extraction form. Data extracted included (1) purpose of intervention, (2) duration of intervention, (3) frequency of text messages, (4) setting, (5) intervention, (6) comparison, (7) one way or two-way SMS, (8) type of participants, (9) follow-up period, (10) outcome results, and (11) reported effect measure (risk ratio, odds ratio, etc.) from the study. The studies included in our updated search were extracted by one reviewer (CL) and 100% checked by a second reviewer (DT). No test of agreement between reviewers was conducted for either of the search phases. All discrepancies were discussed until consensus was achieved.

### Risk of bias assessment

Risk of bias was assessed using Cochrane’s risk of bias (ROB) tool [33]. In Cochrane’s “other” ROB domain, we included “incomplete outcome data (intention to treat [ITT] analysis).” This was defined as randomized participants having been analyzed according to their allocated treatment, irrespective of whether they were eligible, received the allocated treatment, received another treatment, or received no treatment. Study certainty was rated independently by PL and OW, and any discrepancies were discussed until consensus was reached. The studies included from our updated search involved assessment by one reviewer (CL) and checking by a second reviewer (DT). RCTs were considered at low risk of bias if they were rated at low risk for the following domains: random sequence generation, allocation concealment, incomplete outcome data (attrition bias and intention-to-treat [ITT] analysis), and selective reporting bias.

### Unit of analysis issues

As recommended in the Cochrane Handbook [33], we combined multiple intervention arms to overcome unit-of-analysis error and to create a single pair-wise comparison.

### Certainty of the evidence assessment

We graded the overall certainty of evidence using the Grading of Recommendations Assessment, Development and Evaluation (GRADE) approach [30]. GRADE assessments were conducted by a single reviewer. GRADEpro software [34] was used to create Summary of Findings tables for the following primary outcomes: appointment adherence, adherence to ART by MEMS (medication event monitoring system) pill count, and adherence to ART by self-report. The certainty of the evidence was categorized as high certainty, moderate certainty, low certainty, or very low certainty based on a judgment of the confidence in the effect estimate, whether the true effect is likely to be substantially different from the estimate of effect. The certainty of the evidence is downgraded from high, moderate, low to very low for the following reasons: risk of bias, inconsistency (unexplained heterogeneity, inconsistency of results), indirectness of evidence (indirect population, intervention, control, outcomes), imprecision of results (wide confidence intervals), and risk of publication bias.

### Analysis

Clinical heterogeneity was assessed within each pairwise comparison by comparing characteristics [33]. In order to minimize heterogeneity, outcomes were categorized as follows: (1) SMS used for prevention of STIs/HIV, (2) SMS used to impact adherence to HIV medication adherence, and (3) SMS used to impact HIV treatment outcomes. Sub-analyses were conducted based on these different categories. As the characteristics of the 35 included studies were variable, we chose to use the Mantel-Haenszel random effects model. Statistical heterogeneity was examined using the *I*^2^ statistic and interpreted as an *I*^2^ estimate of 0% = no heterogeneity, 25% = low, 50% = moderate, and 75% = high heterogeneity of effect sizes [33]. We summarized dichotomous outcomes using odds ratios (OR), with 95% confidence intervals (CI). We used RevMan 5.3 to conduct the statistical analysis and graphs.

### Publication bias

If more than ten studies reported the same outcome, publication bias was explored with the Egger test and a funnel plot using STATA 13 software with the metan, metafunnel, metabias, and metatrim packages [35–39]. A *p* value less than 0.05 was considered statistically significant. Stata’s trim and fill method was used to estimate the number of missing null studies from the meta-analysis. Stata’s metatrim command performs the Duval and Tweedie nonparametric “trim and fill” method of accounting for publication bias in meta-analysis. The method, a rank-based data augmentation technique, formalizes the use of funnel plots, estimates the number and outcomes of missing studies, and adjusts the meta-analysis to incorporate the theoretical missing studies.

Sensitivity analyses were conducted with and without statistical outliers to assess their effect on the overall findings. In addition, a sensitivity analysis was conducted to assess the impact of RCTs judged to have low methodological certainty on the summary effect. If three or more studies exist in each group, we subgrouped adherence as “self-reported” and “objectively-measured” adherence (MEMS pill count).

## Results

**Fig. 1:**
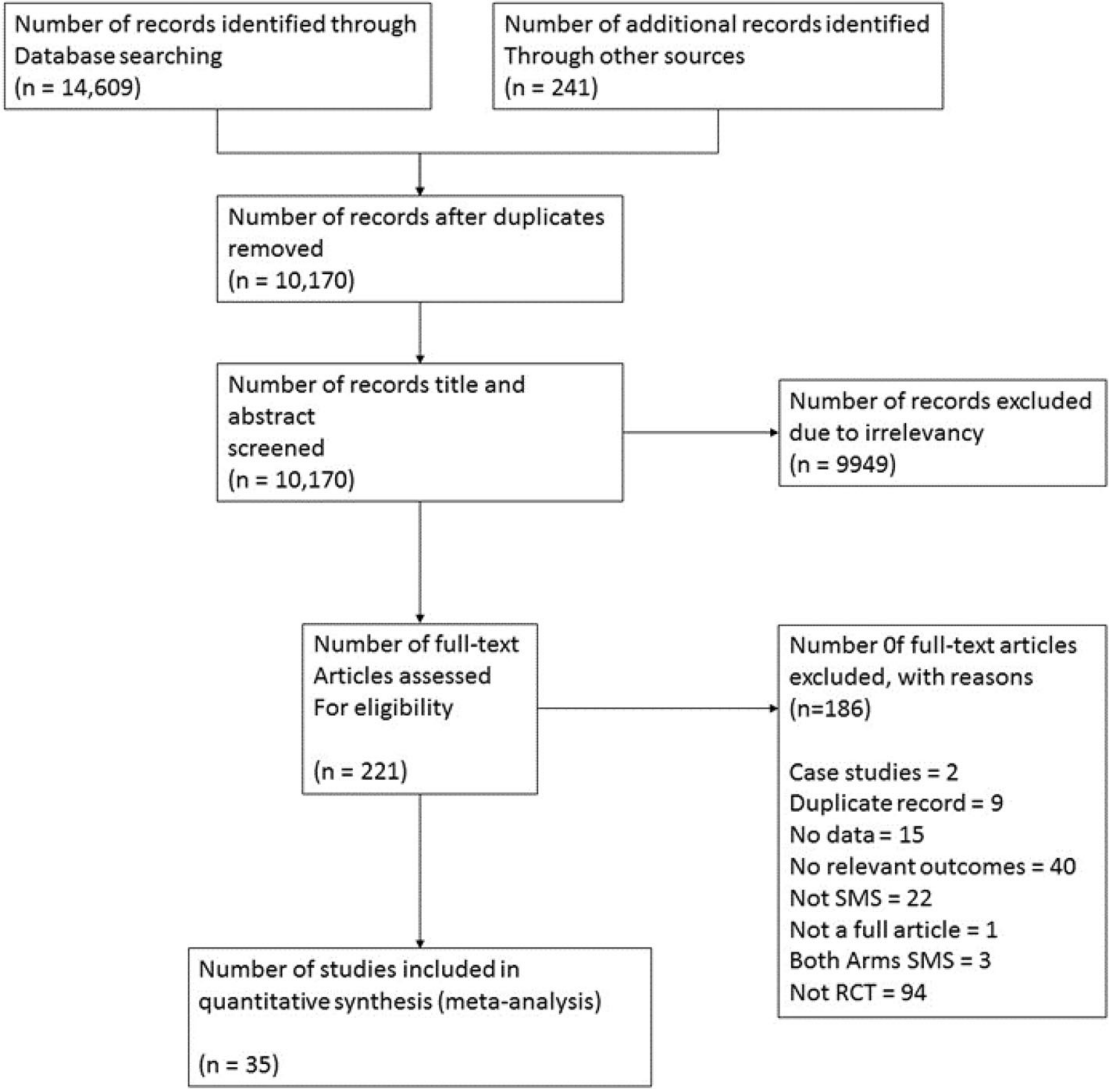
PRISMA flow chart displaying articles included and excluded

A total of 14,850 articles were initially identified through database and manual searches conducted between 1996 and March 2017 (both phases) (Fig. 1). Four thousand six hundred eighty duplicates were removed, and 9949 were not included as they were not relevant to our topic. Of the remaining 221 full text articles, 186 were excluded because they were case studies (*n* = 2), duplicate records of the same study (*n* = 9), included no data and authors were unreachable (*n* = 15), had no relevant outcomes (*n* = 40), did not involve texting (*n* = 22), both arms received text messages (*n* = 3), was not a full article (*n* = 1), and not a RCT (*n* = 94). The remaining 35 RCTs [40–73] were included. Table 1 displays the characteristics of the 35 included studies. A description of the content of the SMS messages can be found in Additional file 5.

**Table 1:** Characteristics of included studies (number of studies = 35)

First author, year	Interv. period	*N*	Country	One-way or two-way SMS	Setting	Intervention	Frequency	Comparison	Participants	Follow-up	Outcome results	Reported effect measure from the study
Barnabas 2016 [40]	June 2013–Mar 2015	750	South Africa and Uganda	One-way	Rural clinic	Promotional text sent after HIV testing to encourage circumcision	One text 3 weeks after testing + 1 phone call 1 month after	Standard clinic referral	Men 16–49 years HIV-negative and uncircumcised	3 months	Uptake of circumcision: proportion being circumcised	RR 1.72 (1.36–2.17)
Bigna 2014 [41]	Jan and May, 2013	121	Cameroon	One-way	Urban (Essos), semi-urban (Kousseri), and rural (Goulfey) hospitals	SMS reminder	2 days prior to appointment	No SMS	Adults ≤ 18 years accompanying an HIV-positive child ≥ 15 years	Unknown	Appointment adherence: proportion attending	OR 2.9 (1.3–6.3)
da Costa 2012 [42]	2008–2009	21	Brazil	One-way	Multidisciplinary Center for Infectious Diseases in Pregnancy, Federal University of São Paulo	SMS reminder 30 min before their last scheduled dose of medicine	Every 2 days	No SMS	HIV-positive Brazilian women	4 months	Medication adherence: MEMS ≤ 95% adherence	NR
Davey 2016 [43]	Nov 2011–Mar 2013	830	Mozambique	One-way	One rural and two urban public health clinic	SMS reminders	2 and 7 days prior to appointment	Standard care	Adults ≤ 18 years receiving first-line ART, for over 15 days	12 months	Appointment adherence: proportion attending	RR 0.68 (0.41–1.13)
de Tolly 2012 [44]	Unknown	2553	South Africa	One-way	General population	3–10 motivational or informative SMS messages	Every 3 days	No SMS	Subscribers of a mobile phone service	3 weeks	Uptake of testing: proportion tested for HIV	OR 1.09 (0.83–1.34)
Downing 2013 [74]	Jan 2010–Mar 2011	94	Australia	One-way	Sexual health clinic	SMS reminder	1 week prior to test of cure	Standard care	Chlamydia + or named as a contact to someone diagnosed with chlamydia	3–4 months	Uptake of testing: proportion who underwent test of cure	OR 5.87 (1.16–29.83)
Dryden-Peterson 2015 [45]	July 2011–April 2012,	366	Botswana	One-way	20 antenatal clinics	Automated platform permitting monitoring and delivery of CD4 results via text	Clinic receipt of results was confirmed centrally via SMS	Standard care	Women with a CD4 count of 250 cells/μL or less were eligible for ART	8 weeks	Uptake of testing: CD4 testing before 26 weeks gestation ART initiation: before 30 weeks gestation	CD4 testing: aOR 0.87 (0.47–1.63) ART initiation: aOR 1.06 (0.53–2.13)
Garofalo 2016 [46]	Oct 2010–Feb 2014	105	USA	Two-way	Research facilities	SMS reminder	Daily for 6 months	No SMS	Poorly-adherent HIV-positive adolescents (aged 16–29)	6 months	Medication adherence: self-reported VAS of 90% adherence Viral load suppression: (< 75 copies/ml)	Adherence: OR 2.12 (1.01–4.45) Viral load suppression: OR 0.77 (0.24–2.49)
Haberer 2016 [47]	Sept 2013-June 2015	63	Uganda	One-way	Mbarara Regional Referral Hospital	Reminder scheduled SMS or triggered SMS	Daily for 1 month then weekly for 2 months	No SMS	HIV-positive individuals taking ART	3 months	Medication adherence: MEMS adherence 100% Viral load suppression: (< 100 copies/ml)	Adherence: IRR 0.6 Viral load suppression: IRR 1.0
Hardy 2011 [48]	Aug 2008–Dec 2008	23	USA	Two-way	Outpatient HIV clinic in Boston	SMS reminders	Daily	One reminder beep at the time of dosing	HIV-positive adults on ART for at least 3 months and reporting less than 85% adherence in past 7 days	6 weeks	Medication Adherence: MEMS at 6 weeks (% not defined)	Adherence (mean difference + [SD]): 33.4 + 9.1
Ignersoll 2015 [49]	May 2012-Aug 2013	63	USA	Two-way	Rural HIV clinic in Virginia	SMS system sent a query, received a response from the participant	Daily: 4 per day	Usual care	HIV-positive adults who reported less than 95% adherence in the past 2 weeks	3 months	Medication adherence: pharmacy refill ≤ 95% adherence Appointment adherence: proportion of missed visits	NR
Kalichman 2016 [50]	Aug 2011–Mar 2015	600	USA	One-way	Infectious disease clinics in Atlanta	SMS reminders for up to 2 daily medication times	Daily: 2 times per day	No SMS	Adults aged 18 or older, on ART	12 months	Medication adherence: medication refill ≤ 95% adherence Viral load suppression: (< 100 copies/ml)	Adherence reported as not significant Viral load suppression: OR 1.24 (1.01–1.52)
Kempe 2016 [51]	Jan 2013–Dec 2013	701	USA	One-way	Pediatric practices in Colorado	Recalling adolescents who were late for HPV doses	Not specified	Standard care	Parents of adolescents aged 11 and 17	Unknown	HPV vaccine series completion: HPV dose 3 completion rates	Vaccine series completion: IRR 1.47 (1.38–1.57)
Lester 2010 [52]	May 2007–Oct 2008	538	Kenya	Two-way	HIV clinic	SMS received from a clinic nurse and response required within 48 h	Weekly	No SMS	Patients initiating ART	12 months	Medication adherence: self-reported ≤ 95% adherence Viral load suppression: (< 400 copies/ml)	Adherence: RR 0.85 (0.72–0.99) Viral load did not achieve suppression level: RR 0·84 (0·71–0·99)
Lim 2012 [53]	Jan 2006–Jan 2007	994	Australia and New Zealand	One-way	Music festival	SMS sexual health promotion messages	Every 3–4 weeks	No SMS	Aged between 16 and 29, and were current residents of Victoria or Tasmania and had a mobile phone number	6 and 12 months	Sexual health behaviour change: Always condom use at 6 months, knowledge of STIs at 6 months Uptake of testing: STI testing at 6 months	Condom use: F: OR 1.34 (0.58–3.09) M: OR 0.97 (0.35–2.66) Knowledge of STIs/HIV: F: OR 2.36 (1.27–4.37) M: OR 3.19 (1.52–6.69) Uptake of testing: F: OR 2.51 (1.11–5.69) M: OR 0.79 (0.22–2.89)
Maduka 2013 [54]	2011	104	Nigeria	One-way	Tertiary hospital	Text about adherence and a reminder to take ART	Twice weekly	Standard Care	HIV positive patients known to be non-adherent to HAART	4 months	Medication adherence: Self-reported ≤ 95 adherence CD4 cell count increase: Median CD4+ cell count	Adherence: RR 0.75 (0.55–0.96) CD4 cell count: intervention increased from 193 cells/ml to 575.0 cells/ml and control from 131.0 cells/ml to 361.5 cells/ml.
Mbuagbaw 2012 [55]	Nov–Dec 2010	200	Cameroon	One-way	Yaounde’ Central Hospital	Weekly motivating text to remind about adherence	Weekly	No SMS	HIV-positive adults on ART, aged 21 years and above	3 months	Medication adherence: Self-reported VAS adherence Presence of new OI	Adherence: RR 1.06 (0.89–1.29) Presence of new OI: OR 1.56 (0.85–2.85)
Moore 2015 [56]	Unknown	50	USA	Two-way	University Research Centre	3 SMS daily	Daily	No SMS	HIV-infected methamphetamine users	30 days	Medication adherence: MEMS adherence	NR
Morris 2015 [57]	Sept 2012–Sept 2013	116,878	USA	One-way	San Diego County Immunization Registry records	Parents chose to receive at least one reminder: mail, e-mail, or text reminder	Every 2 weeks until compliant	Email or phone call	Parents/guardians of 11–17 year old males and females	6 months	HPV vaccine series completion: HPV dose 3 completion rates	NR
Mugo 2016 [58]	April and July 2013	410	Kenya	One-way	Health facilities and community pharmacies	SMS and phone-call reminders	One day prior to appointment	Phone call or in-person reminders	18–29 year old patients	Unknown	Uptake of repeat HIV testing: Proportion attending	NR
Norton 2014 * [59]	June–Aug 2010	52	USA	One-way	HIV clinic	SMS reminder	One prior to appointment	Home phone call	HIV-positive adults aged > 17 years	1 month	Appointment adherence: proportion attending	NR
Nsagha 2016 [60]	Aug–Sept 2011	90	Cameroon	One-way	Hospital	Educative text and standard treatment	Weekly: 4 times a week	Standard care	HIV-positive adults aged 23–62 years	1 month	Medication adherence: self-reported ≤ 95% adherence	NR
Odeny 2012 [61]	Sep 2010–Apr 2011	1200	Kenya	One-way	Circumcision clinic	SMS reminders at the post-operative visit	Daily for 7 days before appointment and then 7 daily post-operative	No SMS	Men undergoing circumcision	7 days	Appointment adherence: proportion attending	Appointment adherence: RR 1.09 (0.99–1.19)
Odeny 2014 [62]	Sep 2010–Apr 2011	392	Kenya	Two-way	Maternal postpartum HIV clinic	Text messages	Daily: 8 texts before delivery and 6 texts postpartum	No SMS	HIV-positive pregnant women at least 18 years old	7 days	Appointment adherence: proportion attending Uptake of testing: virological infant testing by 8 weeks postpartum	Appointment adherence: RR 1.66 (1.02–2.70) Uptake of testing: 92% SMS vs 85.1% control RR 1.08 (1.00–1.16)
Orrell 2015 [63]	July 2012–2014	230	South Africa	One-way	HIV clinic	SMS reminders	One if dosing ≥ 30 min late	No SMS	ART-naive participants	48 weeks	Medication adherence: MEMS medication refill ≤ median adherence Viral load suppression: (≥ 40 copies/mL)	Medication adherence: aOR 1.08 (0.77–1.52) Viral load suppression: aOR 0.77 (0.42–1.40).
Patel 2014 [64]	Sep 2011–Oct 2012	365	USA	One-way	Planned Parenthood health centers	Text messages	Unknown	Standard care	Females 19–26 who were vaccinated once for HPV	32 weeks	HPV vaccine series completion: HPV dose 3 rates	aOR 0.97 (0.55–1.68)
Perron 2010 [65]	Apr–Jun 2008	2123	Switzerland	One-way	Primary care and HIV clinics	Phone, text, mail	One 2 days prior to appointment	No SMS	Adult patients (mean age 46)	36 weeks	Appointment adherence: proportion attending	NR
Pop-Eleches 2011 [66]	Jun 2007–Jan 2008	431	Kenya	One-way	Chulaimbo Rural Health Center	Four different SMS reminder interventions	Daily or weekly	No SMS	Patients who had initiated ART within 3 months	48 weeks	Medication Adherence: MEMS ≤ 90% adherence	NR
Rand 2015 [67]	July 2013–March 2014	1924	USA	One-way	Large not-for-profit MCO	Parents sent reminders of HPV vaccine dose	Up to 4 text messages sent	Standard care	Parents of publicly insured adolescents aged 11–16 years	8 months	HPV vaccine series completion: HPV dose 3 rates	HR 1.30 (0.7–2.6)
Rand 2017 [68]	Dec 2013–April 2014	391	USA	One-way	3 primary care urban clinics in New York	SMS reminder	3 reminders for each dose (1 week apart)	Sent 1 text with a health message	Parents of 11- to 17-year-olds	Unknown	HPV vaccine completion: HPV dose 3 rates	HR 2.34 (1.67–3.27)
Richman 2016 [69]	Aug 2011 Dec 2013	264	USA	One-way	University campus student health center	5 messages and 2 reminders	Monthly: 7 messages, once per month	Paper card with next appointment date	Uni students ages of 18 and 26 voluntarily initiating the first HPV vaccine dose	7 months	HPV vaccine completion: HPV dose 3 rates	HPV vaccine completion not significantly different
Rutland 2012 [70]	ND	252	UK	One-way	GU medicine (GUM) clinics	SMS reminder	SMS sent 1 week after missed appointment	No SMS	Non-attending patients aged 16–30 years	6 months	Appointment adherence: proportion re-attending	NR
Sabin 2015 [71]	Dec 2012–Oct 2013	119	China	One-way	Guangxi Center for Disease Control and Prevention ART clinic	Reminders after late dose taking	Unlimited	No SMS	HIV-positive adult patients on HIV treatment	6 months	Medication adherence: MEMS ≤ 95% optimal adherence	Optimal adherence: RR 1.7 [1.3–2.2]
Shet 2014 [72]	July 2010–June 2011	631	South India	Two-way	Hospitals in Bangalore, Mysore, and Chennai	Pictorial reminder 4 days after an automated motivationalcall	Weekly	No SMS	Patients on ART	24 months	Medication adherence: medication refill ≤ 95% adherence Viral load suppression: (> 400 copies/mL)	Suboptimal adherence: IRR 1.24 (0.93–1.65) Viral load did not achieve suppression levels: HR 0.98 (0.67–1.47)
Suffoletto 2013 [73]	Sep 2011–Apr 2012	52	USA	Two-way	Urban emergency department	SMS behavioral questions	Weekly	SMS reminders to complete study questionnaires	Female patients aged 18–25 years with hazardous drinking behavior and recent risky sexual encounters	3 months	Sexual health behaviour change: always condom use in past 28 days; alcohol or drug use in past 28 days (not reported)	Sexual health behaviour change: OR 1.32 (0.31–5.71)

*aOR* adjusted odds ratio, *ART* antiretroviral therapy, *GUM* genitourinary medicine, *HPV* human papillomavirus, *HR* hazard ratio, *Interve*: intervention, *IRR* incidence rate *ratio*, *MCO* managed care organization, *MEMS* medication event monitoring system, *mL* milliliter, *NR* not reported, *OI* opportunistic infections, *OR* odds ratio, *RR* risk ratio, *SD* standard deviation, *SMS* short message service, *VAS* visual analog scale

*Recruited participants. While they were in the clinic, we were already selecting for a group of patients who were more likely to attend their next appointment

**Nonattenders such as at highest risk for nonattendance

Of the 35 studies included in this systematic review, the majority investigated either the effectiveness of text messaging to support STI clinic appointment attendance (8 studies), or the effectiveness of text messaging to support HIV medication adherence (15 studies). These studies were conducted in 14 countries, the majority being in the USA (13 studies), Kenya (5 studies), and South Africa (3 studies) and were conducted in a variety of settings including clinics (19 studies), hospitals (6 studies), universities (4 studies), and others such as an emergency room (1 study) and a music festival (1 study). The age of participants ranged from 16 to 84 years and the length of follow-up ranged from 7 days to 24 months. Text messages were either sent daily, weekly, monthly, or as one-off reminders. The majority of studies (83%) sent a one-way text with the remaining sending two-way texting.

### Risk of bias across studies

The majority of studies (88.6%) had low risk of random sequence generation using either minimisation, varying block size or computer-assisted randomization. Four studies had unclear risk of random sequence generation [48, 56, 69, 70]. One study was at high risk of allocation concealment [18], 13 studies were at unclear risk [45, 47, 51, 56, 57, 59, 60, 64, 65, 68–70, 73], and the 21 remaining studies were at low risk.

In most studies, medical staff were masked to the knowledge of the allocated interventions; however, in the majority of cases, blinding of participants to intervention allocation was not done. Only two studies successfully blinded participants to the intervention [57, 67]. Participants in the Morris et al. [57] study were from a registry so they were blinded to their randomization group, and individuals in Rand et al.’s [67] control group received the same initial message as the intervention group, followed by a control message about a different general adolescent health topic each time reminders were sent to the intervention group; therefore, they were unlikely to ascertain their allocation.

High attrition bias was encountered in three studies [40, 56, 73] and was unclear in three others [45, 46, 70] as they had not recorded the number of participants lost to follow-up. Thirteen studies were at high risk of incomplete outcome bias (intention-to-treat [ITT] analysis) as they had not conducted the ITT analysis correctly or at all [40, 42, 44, 45, 48, 49, 53, 56, 57, 61, 62, 69, 73]. An intention-to-treat (ITT) analysis is recommended as the least biased way to estimate intervention effects in randomized trials [33].

**Fig. 2:**
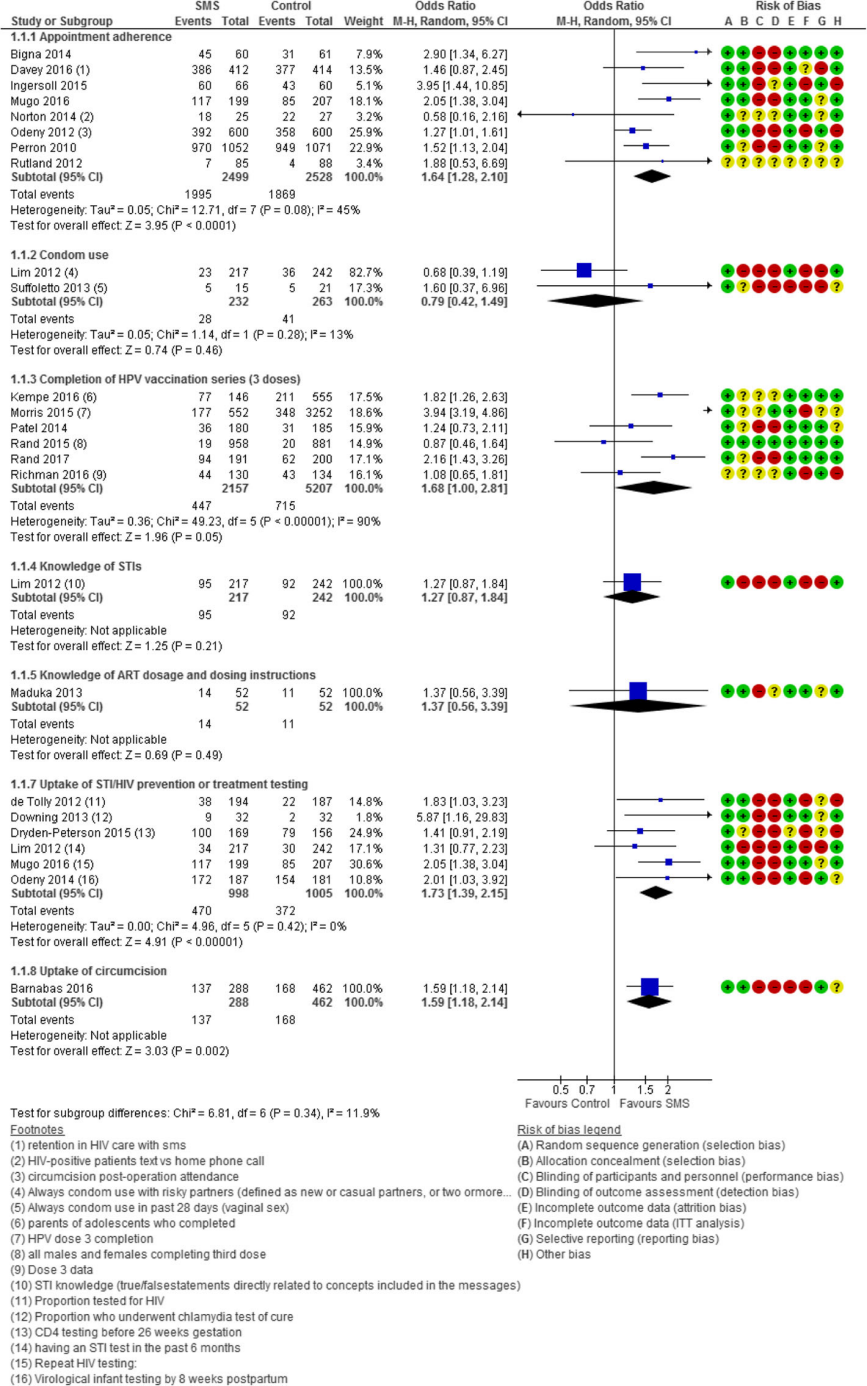
Forest plot of the effectiveness of text messaging on STI/HIV prevention

**Fig. 3:**
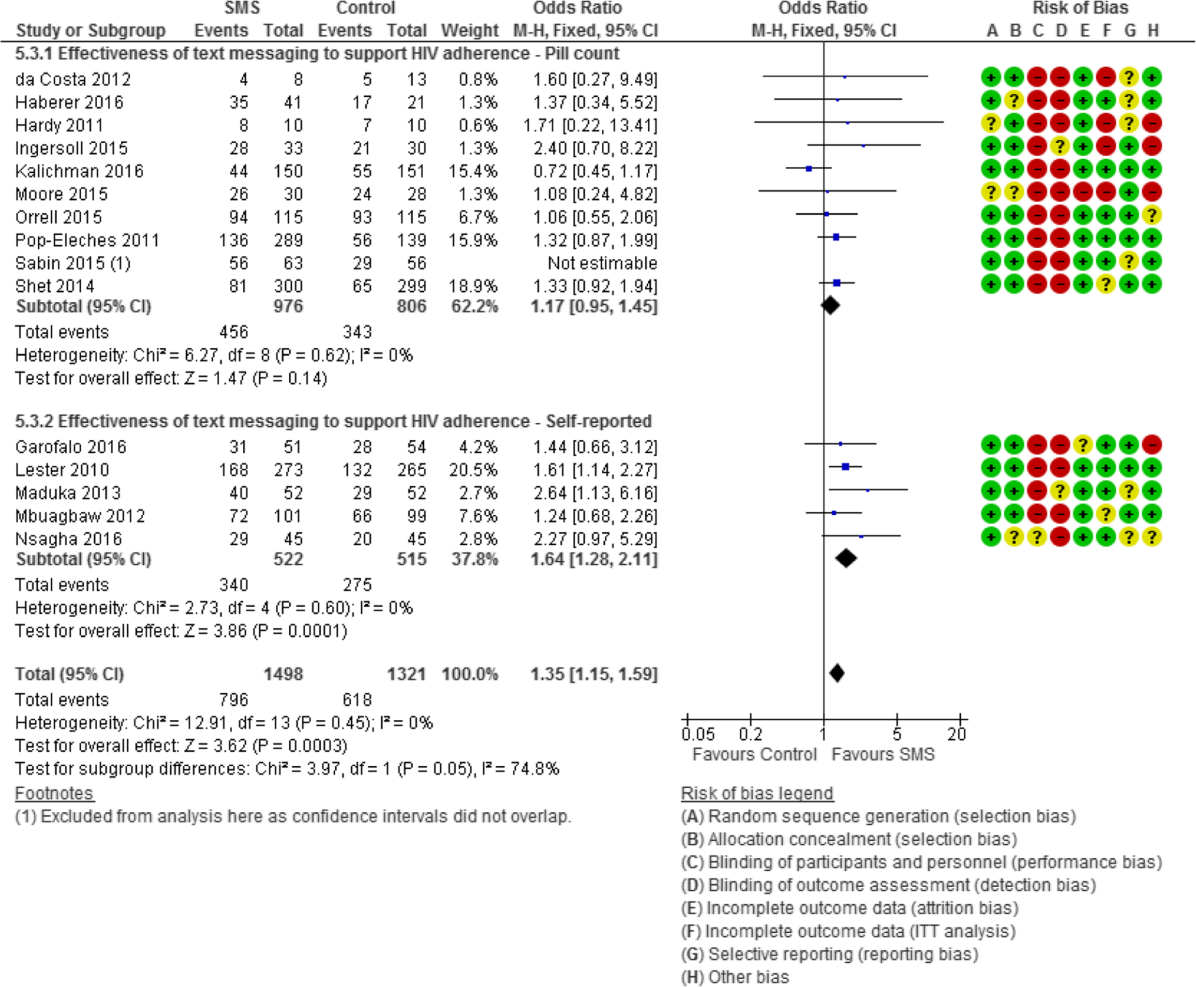
Forest plot of the effectiveness of text messaging on HIV treatment adherence

**Fig. 4:**
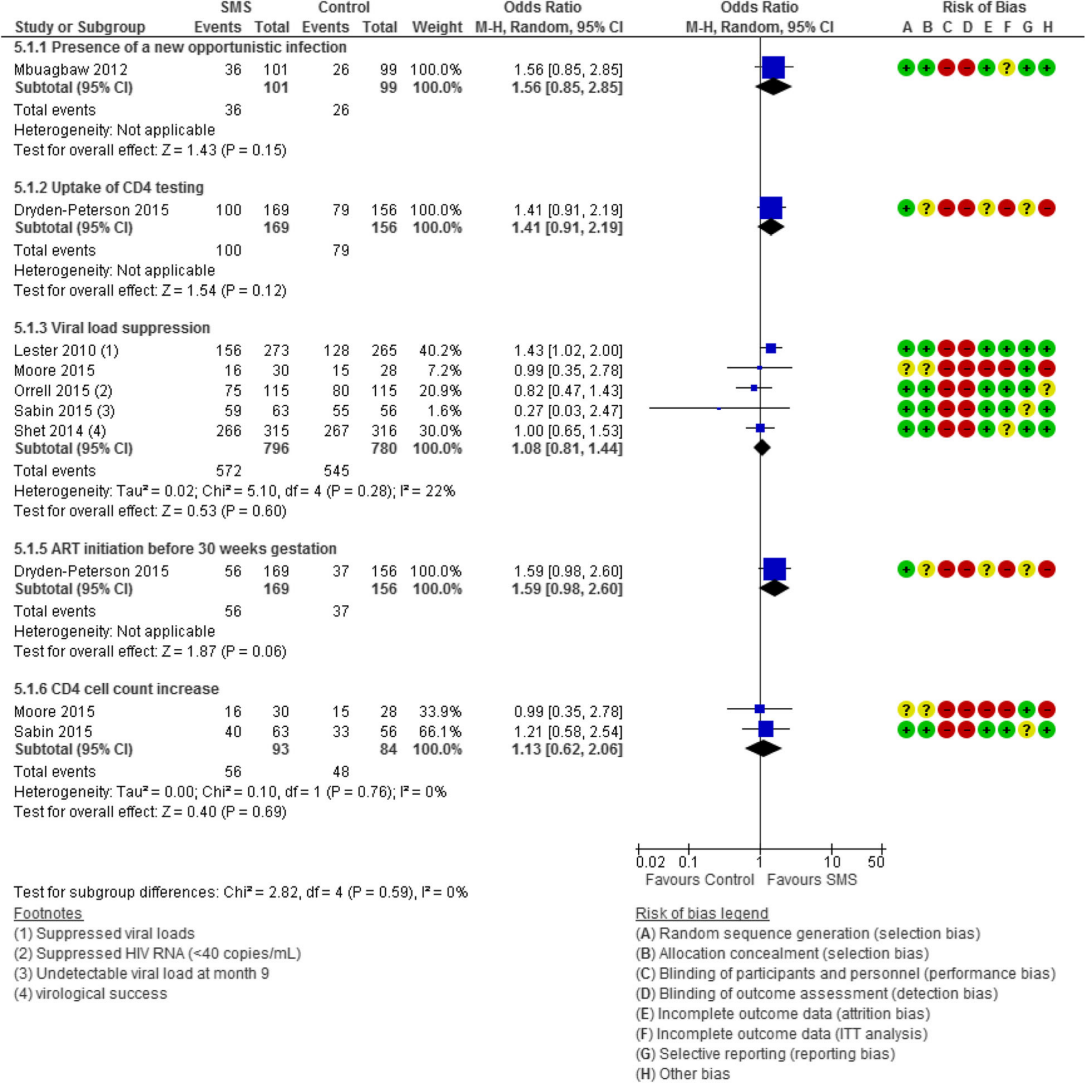
Forest plot of the effectiveness of text messaging on HIV treatment outcomes

Selective reporting bias was at high risk in three studies [43, 53, 73] and at unclear risk in eight studies [42, 44, 45, 47, 48, 54, 71, 74] because of pre-protocol or registry information being unavailable. Six studies were considered at low risk of bias as they were rated at low risk for the following domains: random sequence generation, allocation concealment, incomplete outcome data (attrition bias and ITT analysis bias), and selective reporting bias [41, 50, 52, 63, 66, 68]. Only three of these measured the same outcome (HIV adherence by pill count) and were therefore available for sensitivity analysis [50, 63, 66]. Risk of bias for each study is included in the forest plots provided (Figs. 2, 3, and 4). In addition, a summary graph of the risk of bias for all included studies and a detailed graph of the risk of bias of individual studies can be found in Additional file 6.

### Outcomes

Outcomes included in our analysis were divided into three categories: prevention interventions, HIV drug adherence interventions, and HIV treatment outcome interventions. A table organizing studies for subgroup analysis can be found in Additional file 7.

#### Prevention interventions (Fig. 2)

##### Appointment adherence

Eight studies [41, 43, 49, 58, 59, 61, 65, 70] with a total of 5027 participants found an associated increase in appointment attendance among the SMS group compared to standard care (OR 1.64, 95% CI 1.28 to 2.10). There was low heterogeneity among the pooled studies (*I*^2^ 45%). Two studies that recruited youth (age 16–30 years) had a pooled odds ratio of 2.03 with a wide confidence interval of 1.39 to 2.97 indicating imprecision [58, 70]. In the case of Rutland et al.’s study [70], imprecision may have been a result of a small sample size.

##### Condom use

Two studies [53, 73] with 1046 participants (aged between 16 and 29) examining the effectiveness of SMS on condom use reported no association between the intervention and control groups (OR 0.79, 95% CI 0.42 to 1.49). This pooled effect was powered by Lim et al.’s study [53] (*n* = 994 enrolled; 54% attrition) that indicated condom use did not increase in the treatment group at 6 months follow-up compared to Suffoletto et al.’s study [73] (*n* = 52 females; 30% attrition) that reported a positive effect of the SMS intervention at 3 months follow-up. There was low statistical heterogeneity (*I*^2^ 13%) among the two studies; however, the two studies differed in the type of participants recruited and outcome definition. Lim et al. [53] recruited males and females at a music festival and defined condom use as always using condoms in the past 3 months with risky partners. In contrast, Suffoletto et al. [73] recruited females with hazardous drinking behavior and recent risky sexual encounters and defined condom use as always using condoms in the past month.

##### Uptake of STI/HIV testing

Pooled results from six studies (*n* = 4753; 58% attrition) revealed a positive association between SMS and uptake of STI/HIV testing (OR 1.73, 95% CI 1.39 to 2.15; *I*^2^ 0%). Two studies [44, 58] evaluated the effectiveness of SMS on the uptake of HIV testing compared to standard care, one study [53] examined the effectiveness of SMS on the uptake of STI testing in the past 6 months compared to standard care, one study [74] examined those undergoing chlamydia test of cure, and two others examined CD4 testing before 26 weeks gestation [45] and viral load testing in infants by 8 weeks postpartum [62].

##### HPV vaccination series completion

Six studies [51, 57, 64, 67–69] (*n* = 10,301; 29% attrition) examined the effectiveness of text SMS compared to no reminders on completion of a three-dose HPV series. The pooled comparison found a non-significant association (OR 1.68, 95% CI 1.00 to 2.81) with high heterogeneity (*I*^2^ 90%). One study by Morris et al. (OR 3.94, 95% CI 3.19 to 4.86) was a clear outlier as its confidence intervals did not overlap with the other five studies, and when it was taken out of the pooled analysis, heterogeneity lowered to *I*^2^ 55% and the confidence interval around effect estimate became more precise (OR 1.43, 95% CI 1.04 to 1.97; Fig. 2). Two studies with populations aged between 18 and 26 were subgrouped and produced a pooled odds ratio of 1.16 (95% CI 0.86 to 1.67) indicating no association between the intervention and control groups.

##### Knowledge of STIs, ART dosage, and instructions

Two studies [53, 54] evaluated the effect of SMS on education surrounding STIs [53] and ART dosage and instructions [54] (*n* = 1098; 49% attrition). Neither study showed a positive effect. Participants of the Maduka and Tobin-West study [54] did not have a statistically significant increase in knowledge of ART dosing and dosing instructions (OR 4.55, 95% CI 0.92 to 22.55), nor with drug names (OR 1.37, 95% CI 0.39 to 3.39). However, the wide confidence interval (due to small sample size) in the knowledge of ART dosing and dosing instructions outcomes indicates substantial imprecision.

##### Circumcision

One study [40] that examined the effectiveness of sending promotional texts to HIV-negative and uncircumcised men after HIV clinic testing to encourage circumcision compared to standard clinic referral found a positive association (OR 1.59, 95% CI 1.18 to 2.14). However, another study of men who underwent circumcision [61] reported no association between men who resumed sex before the waiting period that was recommended by their doctor in the SMS group and the control group (RR 1.09, 95% CI 0.99 to 1.19).

#### Adherence to antiretroviral therapy (Fig. 3)

Fifteen RCTs (*n* = 2819) examined the effectiveness of text messaging to support increased antiretroviral therapy (ART) adherence compared to standard care (no text). Ten of these studies measured adherence objectively using pill counts [42, 47–50, 56, 63, 66, 71, 72], and five used self-reported adherence [46, 52, 54, 55, 60].

Overall, the pooled 15 text message interventions found a positive association with increased adherence compared to standard care (OR 1.43, 95% CI 1.22 to 1.67; *I*^2^ 44%). When an outlier, Sabin et al.’s study [71], was omitted from overall pooled analysis (14 studies), the analysis became homogenous and the association remained positive albeit weaker (OR 1.35, 95% CI 1.15 to 1.59; *I*^2^ 0%). However, in subgroup analysis, the ten studies measuring adherence by pill count (MEMS) (omitting [71]) found a non-significant association with ART adherence (OR 1.17, 95% CI 0.95–1.45) compared to the significant association among the five studies measuring adherence by self-report (OR 1.64, 95% CI 1.28–2.11). When the three studies at low risk of bias were pooled in subgroup analysis [50, 63, 66], there was no association found between the text messaging arm and the standard care arm (OR 1.03, 95% CI 0.78–1.37; *I*^2^ 42%).

The five RCTs measuring adherence by self-report were homogenous. Heterogeneity in the ten studies measuring adherence by pill count (*I*^2^ 56%) was moderate when all studies were combined. However, when the outlier study by Sabin et al. [71] (OR 7.45, 95% CI 2.90 to 19.15) was omitted, the RCTs became homogenous (*I*^2^ 0%), and the effect measure weakened to show no association (OR 1.17, 95% CI 0.95 to 1.45).

#### HIV treatment outcomes (Fig. 4)

##### Uptake of CD4 testing

One study by Dryden-Peterson et al. (*n* = 366; 11% attrition) [45] examined the effectiveness of text messaging on the uptake of CD4 cell count testing revealing no significant association (OR 1.41, 95% CI 0.91 to 2.19).

##### Viral load suppression

Five studies (*n* = 1607; 2% attrition) examined the effectiveness of SMS to support HIV viral suppression [52, 56, 63, 71, 72]. In pooled analysis, the five RCTs showed no association in virological suppression below the level of detection between intervention and control groups with low heterogeneity (OR 1.08, 95% CI 0.81 to 1.44; *I*^2^ 22%).

##### Presence of a new opportunistic infection

One study [75] (*n* = 200; 0% attrition) reported the presence of new opportunistic infections in HIV positive participants at 3 months post-intervention. This study showed that SMS was not associated with the incidence of new opportunistic infections (OR 1.56, 95% CI 0.85 to 2.85).

##### CD4 cell count increase

Two studies (*n* = 208; 15% attrition) [56, 71] examining the proportion of patients that had an increase in CD4 cell count when a text message was received found no association (OR 1.13, 95% CI 0.62 to 2.06; *I*^2^ 0%). In addition to these two studies, Maduka (*n* = 104) [54] measured pre- and post-intervention median CD4 cell counts among intervention and control groups and found a greater increase in CD4 cell counts in the text message group than the control group (SMS group increased from 193 cells/ml to 575.0 cells/ml compared to the control group that saw an increase from 131.0 cells/ml to 361.5 cells/ml; *p =* 0.007).

##### Uptake of CD4 testing

One study (*n* = 366; 11% attrition) [45] examined the uptake of CD4 testing when SMS were sent to the intervention group compared to standard care found no association (OR 1.41. 95% CI 0.91, 2.19).

##### ART initiation before 30 weeks gestation

One study (*n* = 366; 11% attrition) [45] examining text messaging to support ART initiation before 30 weeks gestation showed no association between the intervention and standard care groups (OR 1.59, 95% CI 0.98 to 2.60).

### Test of publication bias

Medication adherence was the only outcome that had sufficient studies to conduct a test of publication bias. The funnel plot with the trim and fill method (Additional file 7) for the 15 medication adherence studies indicated asymmetry. The Egger test of small-study effects revealed an estimated bias coefficient of 1.53 (standard error 0.41; *p* = 0.002). The trim and fill method predicted that two RCTs were missing from this analysis.

### GRADE assessment

We assessed all outcomes using the GRADE approach. Results for three of the most important outcomes are in Table 2. The remaining assessments are available in Additional file 8. The eight RCTs contributing data to the appointment adherence outcome showed serious risk of bias [41, 43, 49, 58, 59, 61, 70]. The majority of studies were single-blinded; however, the outcome assessment appeared free from bias because the investigators blinded outcome assessors. There was a lack of blinding of patients in studies, and only one study clearly used intention to treat analysis. Inconsistency and indirectness were assessed as serious because of moderate heterogeneity of effect estimates, and populations varied significantly. Imprecision was considered serious because of wide confidence intervals. Publication bias was strongly suspected. Appointment adherence was therefore assessed as very low-certainty evidence.

**Table 2:** GRADE assessment of three important outcomes: appointment adherence, antiretroviral adherence by pill count and self-report

Certainty assessment							No. of patients		Effect		Certainty	Importance
No. of studies	Study design	Risk of bias	Inconsistency	Indirectness	Imprecision	Other considerations	Text messaging	Control	Relative (95% CI)	Absolute (95% CI)		
Effectiveness of text messaging to support STI prevention and treatment interventions—appointment adherence												
8^a^	Randomized trials	Serious	Serious^b^	Serious^c^	Serious	Publication bias strongly suspected	1995/2499 (79.8%)	1869/2528 (73.9%)	OR 1.64 (1.28 to 2.10)	84 more per 1000 (from 45 more to 117 more)	Very low	Important
Effectiveness of text messaging to support HIV adherence–effectiveness of text messaging to support HIV adherence-pill count												
10	Randomized trials	Serious^d^	Serious^e^	Serious^f^	Not serious	None	512/1039 (49.3%)	372/862 (43.2%)	OR 1.31 (1.06 to 1.60)	67 more per 1000 (from 14 more to 117 more)	Very low	Important
Effectiveness of text messaging to support HIV adherence–effectiveness of text messaging to support HIV adherence-self-reported												
5	Randomized trials	Serious^g^	Not serious	Serious^h^	Serious^i^	None	340/522 (65.1%)	275/515 (53.4%)	OR 1.64 (1.28 to 2.11)	119 more per 1000 (from 61 more to 173 more)	Very low	Important

*CI* confidence interval, *OR* odds ratio

^a^Serious risk of bias. Although all studies were single-blinded, the outcome assessment appeared free from bias because the investigators blinded outcome assessors. We downgraded because of the lack of blinding of patients in studies and only one study clearly used intention to treat analysis

^b^Serious inconsistency. There was unexplained inconsistency and moderate *I*^2^ values with no statistically significant heterogeneity of effect estimates; however, the confidence intervals did overlap

^c^Serious indirectness. One trial included children only, one trial adult soldiers, and one trial adults and children (> = 14 years). The effect in adults living in the community may be different

^d^Serious risk of bias. We downgraded because 7 out of 10 RCTs were at high risk of bias

^e^Serious inconsistency. There was unexplained inconsistency and moderate *I*^2^ values with statistically significant heterogeneity of effect estimates. One study’s confidence intervals did not overlap ([71])

^f^Serious indirectness. Populations varied, as well as frequency of text reminders sent

^g^Serious risk of bias. One study had unclear risk of allocation concealment. Four out of five RCTs did not have adequate blinding of personnel to the study arms

^h^Serious indirectness. Populations varied, as well as frequency of text reminders sent

^i^Serious imprecision. Wide confidence intervals present

The ten studies contributing data to the adherence to ART by pill count outcome showed serious risk of bias [42, 47–50, 56, 63, 66, 71, 72]. Seven out of ten RCTs were assessed at high risk of bias due to unclear randomization and allocation concealment. There was unexplained inconsistency, with moderate heterogeneity, and one study’s confidence intervals did not overlap [71]. Indirectness was assessed as serious because populations studied varied, as well as the frequency of text reminders sent. Adherence to ART by pill count was therefore assessed as very low-certainty evidence.

The five RCTs contributing data to the adherence to ART by self-report outcome showed serious risk of bias [46, 52, 54, 55, 59] due to the unclear risk of allocation concealment (one study) and inadequate blinding of staff to the study arms (four out of five studies). Inconsistency was not serious as studies showed statistical homogeneity. Indirectness was serious because populations varied across studies, as did the frequency of text reminders sent. There was also serious imprecision because of wide confidence intervals. The adherence to ART by self-report outcome was therefore assessed as very low-certainty evidence.

### Deviations from planned protocol

In our published protocol [31], we stated we would include randomized and nonrandomized controlled trials, pre- and post-test designs, observational (cross-sectional, case-series, case studies, and qualitative studies) that examined the effectiveness of SMS interventions on STIs. However, we only include RCTs in order to manage the large amount of data collected and because these represent the highest quality research. Similarly, we only reported on the 14 outcomes that were most dominant in the literature aimed at assessing the effectiveness of interventions (not qualitative outcomes such as acceptability). We also made our eligibility criteria broader than our originally planned criteria to allow for all types of comparisons (e.g., phone, emails) to be inclusive of other means of communicating health messages.

The initial search was conducted in 2013 in multiple databases (MEDLINE, ACP Journal Club, Database of Abstracts of Reviews, EMBASE, EBM Reviews, and Cochrane Library); however, the update search was conducted only in MEDLINE and mhealthevidence.org to contain the search results due to restricted resources available (staff and funding). We feel however that searching mhealthevidence.org in addition to MEDLINE would have retrieved the majority of randomized trials on this topic as it is a current database repository for all mHealth evidence implemented by Johns Hopkins Center for Communication Programs. Furthermore, in our more comprehensive search from 1996 to 2013 of five databases, we retrieved only 14 RCTs as compared to the additional 21 we retrieved from August 2013 to end of March 2017 (with consideration of the probable increase in SMS studies published) using the MEDLINE and mhealthevidence.org databases.

## Discussion

### Summary of main results

The purpose of this systematic review was to determine the effectiveness of SMS on STI/HIV prevention and control outcomes. Overall, the results of our systematic review highlight the equivocal nature of the evidence surrounding the effectiveness of SMS interventions of STI/HIV outcomes. SMS interventions vary greatly in different populations and when used for different purposes. SMS may be effective for some prevention interventions such as those aimed at improving appointment adherence (eight studies) and those aimed at increasing the uptake of circumcision (to prevent HIV) (two studies). They may also be effective in increasing adherence to HIV medications(15 studies) although this effect was primarily found in studies depending on self-report suggesting that social desirability bias may have come into play. Conversely, pooled results from seven studies indicate that there is no statistically significant association between providing SMS interventions and improving HIV treatment outcomes.

It should be noted that the certainty of the evidence was found to be low due to inconsistency, indirectness, and imprecision. This may be explained by the markedly variant in terms of SMS methods (one-way versus two-way, frequency of text messages sent, their content, and the populations targeted) among the studies we examined. This explanation is supported by others who have experienced disparate effects of SMS using identical study designs, geographic location, and SMS mode of delivery but among a slightly different population group [76]. Moreover, uncertainty among our results may be due to the high percentage of studies (75%) having high risk of selection bias and performance bias due to inability to conceal randomization allocation and inability to blind study participants and study personnel.

### Significance of this review

Our study points out that the evidence related to SMS interventions remains equivocal and individual studies should be interpreted with caution. Randomized controlled trials are considered the gold standard for determining the effectiveness of an intervention. However, when bias is introduced by lack of blinding, small sample sizes, high attrition rates, and unclear allocation concealment, confidence in the evidence is reduced. Our study highlights the challenges that are encountered when attempting to pool results from RCTs that are prone to bias. In addition, there is a need for standardized reporting, using common definitions for outcomes for RCTs aimed at evaluating SMS to reduce unexplained inconsistencies. Finally, we recommend authors of RCT studies to include statements about registration of study protocols in their published work to minimize concerns of reporting bias and include intention-to-treat analyses in their research to avoid bias resulting from crossover.

### Agreements and disagreements with other studies or reviews

Our results are consistent with the findings of a 2013 Cochrane review (*n* = 8) that showed low to moderate certainty related to SMS improving attendance rates at any healthcare appointments [77]. Our findings also agree with a 2017 network meta-analysis [29] reporting results of a pairwise direct comparison of HIV treatment adherence using a SMS intervention versus standard care in four RCTs (OR of 1.70) (95% CI 1.16, 2.49, certainty of the evidence, moderate). Another 2012 Cochrane review (*n* = 2) evaluated SMS for promoting adherence to antiretroviral therapy in patients with HIV conducted in Kenya only [27]. Our analysis included these two high-certainty studies and included an additional 13 RCTs.

Two other reviews pooled the results of RCTs and quasi-experimental controlled trials on the effectiveness of SMS interventions on HIV adherence and viral load [26, 28]. One systematic review with meta-analysis by Mayer and Fontelo examined (1) appointment non-attendance and (2) HIV adherence when an SMS reminder was sent to participants in intervention studies compared to a control group or pre-intervention group [28]. They included both pre-post studies and randomized controlled trials but did not include a subgroup analysis by study design. The pooling of the Mayer and Fontelo data on non-attendance is somewhat counterintuitive as clinicians, researchers, and funders want to know if the addition of SMS reminders will increase appointment attendance at STI/HIV clinics. Further, Mayer and Fontelo’s pooled analysis of the effectiveness of SMS reminders to improve medication adherence was done using a standardized mean difference. The majority of studies in our analysis (67%, *n* = 10/15) reported data as proportion of patients 90–95% adherent compared to control; therefore, we chose to analyze the HIV adherence data on a dichotomous scale. This decision, as well as the decision to pool only RCTs, resulted in a lower level of heterogeneity (*I*^2^ 44% over the 15 RCTs) in our pooled analysis compared to Mayer and Fontelo’s [28] high heterogeneity (*I*^2^ 99%). When performing sensitivity analyses, we omitted the one study that differed in its definition of adherence (patients were categorized as optimally and suboptimally adherent) which resulted in a homogenous analysis (*I*^2^ 0%) and an OR of 1.35 with a narrow confidence interval (95% CI 1.15–1.59).

Two other previous reviews are available. Lim et al. [78] provided a review of SMS interventions. However, this review is outdated (2008) and was limited to describing SMS programs. The authors acknowledge that the programs the report on were not rigorously evaluated. We are providing more current evidence that has been rigorously produced. Zou et al. [79] provided a review that is 4 years old and had a narrow scope. They evaluated the effect of SMS on screening rates for bacterial STIs among men who have sex with men. Our study provides a more comprehensive review of all STIs for all populations. The reference list for this review was utilized during our search strategy.

Systematic reviews by Chavez et al. [80], L’Engle et al. [24], and Zou et al. [81] present important findings with respect to text messaging and sexual health outcomes; however, they do not cover the effectiveness of SMS interventions for a broad spectrum of STI prevention and control strategies. Our review fills an important gap by including more RCTs. In addition, the scope of our review is wider because it provides evidence from developed and developing countries.

### Strengths and limitations of the present review

A major strength of this present review is that we have included an evaluation of SMS for both STIs and HIV. This is important because public health clinicians do not separate out interventions based on these two things. Introduction of bias in our review was minimized by double independent screening and extraction, and assessing the risk of bias of each included study by two authors. Although no test of agreement was conducted between reviewers, all discrepancies were discussed and resolved.

The authors recognize that limiting our review to studies published in English may produce an unintended bias as SMS interventions conducted in non-English-speaking countries may have a different effect. Therefore, we caution readers from non-English speaking countries to take this into consideration when considering introducing SMS interventions in their respective jurisdictions. We did not include studies that could only be found in the grey literature (i.e., conference proceedings) as it is difficult to assess certainty and bias for these studies. This may have resulted in missing more recent studies which may have resulted in including studies with a greater effect size (due to possible increased comfort levels with text messaging). In addition, only one reviewer was involved in searching for studies and screening titles and abstracts for eligibility during phase 2. This may have resulted in some relevant studies spuriously being excluded. Moreover, GRADE assessments were conducted by a single reviewer, due to resource limitations, which may have biased the reporting in an unintended way. In addition, we did not report the results of one primary outcome—acceptability of SMS for STI interventions and one secondary outcome—feasibility of program delivery, which was reported by some RCTs in our sample. We felt that this information was better suited to a subsequent (second paper) reporting implementation results.

The initial search was conducted in 2013 in multiple databases (MEDLINE, ACP Journal Club, Database of Abstracts of Reviews, EMBASE, EBM Reviews, and Cochrane Library); however, the update search was conducted only in MEDLINE and mhealthevidence.org to contain the search results due to restricted resources available (staff and funding). We feel however that searching mhealthevidence.org in addition to MEDLINE would have retrieved the majority of randomized trials on this topic as it is a current database repository for all mHealth evidence implemented by Johns Hopkins Center for Communication Programs. Furthermore, in our more comprehensive search from 1996 to 2013 of five databases, we retrieved only 14 RCTs as compared to the additional 21 we retrieved from August 2013 to end of March 2017 (with consideration of the probable increase in SMS studies published) using the MEDLINE and mhealthevidence.org databases.

The studies we included were of varying certainty, and the certainty of the evidence on two important outcomes was considered low, thus limiting the confidence in the summary effect estimates. We were also only able to pool a sufficient number of high-certainty studies for one outcome—adherence to ART. Both certainty of evidence and study quality were considered when making conclusions, reducing the influence of lower certainty studies. In addition, rigorous searching of multiple databases was conducted; however, publication bias is suspected due to non-publication of many negative trials.

### Future research

High certainty RCTs are required to adequately assess the effect of text messaging to support all STI/HIV prevention and treatment outcomes included in this review. Ongoing trials into text messaging support to STI/HIV interventions might provide more power to update of these meta-analyses [82, 83]. Research into messaging interventions should also include other messaging platforms including WhatsApp, Facebook, etc. and compare results with cell phone texting. Moreover, our study did not report reported adverse events related to the SMS interventions. This should be included in a future review. Future research should also examine whether reminders alone are as effective as reminders embedded in educative messages [84, 85]. Finally, RCTs can be designed as double-blinded by delivering the same number of text messages but with different content to the patient and control groups, thus making the trials less prone to biased results.

### Application to clinical care

SMS is a flexible method to deliver health messages as it allows for instantaneous delivery directly to individuals at any time, place, or setting via their phones [86]. However, it is important to evaluate the effectiveness of an intervention once it has been initiated. The use of digital technology in designing interventions aimed at improving health outcomes including those related to STI and HIV has been approached with great enthusiasm. However, as others have pointed out [80], digital technology is rapidly changing and therefore research that has been conducted with SMS interventions may soon only be useful for a short period of time.

## Conclusion

The effectiveness of SMS interventions to improve STI/HIV outcomes remains equivocal, and due to the lack of precision of our pooled results and inconsistency of findings due to patient characteristic variability, it is incumbent upon program planners to evaluate the effectiveness of any program to ensure it is achieving the intended result.

## Additional files

Additional file 1: PRISMA Checklist. (DOCX 30 kb)

Additional file 2: Ovid Search Strategy Phase 1. (DOCX 17 kb)

Additional file 3: Ovid Search Strategy Phase 2. (DOCX 18 kb)

Additional file 4: Eligibility Criteria. (DOCX 14 kb)

Additional file 5: Table of SMS Messages. (DOCX 21 kb)

Additional file 6: Risk of Bias Figures. (DOCX 47 kb)

Additional file 7: Publication Bias Analysis and Subgroup Analysis Table. (DOCX 26 kb)

Additional file 8: GRADE Assessment of other Outcomes. (DOCX 18 kb)

## Abbreviations

aOR: Adjusted odds ratio
ART: Antiretroviral therapy
CI: Confidence interval
GRADE: Grading of Recommendations Assessment, Development and Evaluation
GUM: Genitourinary medicine
HIV: Human immunodeficiency virus
HPV: Human papillomavirus
HR: Hazard ratio
Interve.: Intervention
IRR: Incidence rate *ratio*
ITT: Intention-to-treat
MCO: Managed care organization
MEMS: Medication event monitoring system
MSM: Men who have sex with men
NR: Not reported
OI: Opportunistic infections
OR: Odds ratio
PICO: Population, Intervention, Comparison, Outcome
PID: Pelvic inflammatory disease
RCTs: Randomized controlled trials
RR: Risk ratio
SD: Standard deviation
SMS: Short message service
SR: Systematic review
STI: Sexually transmitted infection
VAS: Visual analog scale
