# Supplementary material for: Effectiveness of text messaging interventions on prevention, detection, treatment, and knowledge outcomes for sexually transmitted infections (STIs)/HIV: a systematic review and meta-analysis
Source: Syst Rev. 2019 Jan 8;8:12. doi: 10.1186/s13643-018-0921-4 (PMC6323863; doi:10.1186/s13643-018-0921-4)
Supplement: Supplementary file 2 — Ovid Search Strategy Phase 1. (DOCX 17 kb) [file 13643_2018_921_MOESM2_ESM.docx]

**Supplementary file 2: Ovid Search Strategy Phase 1**

Databases: Cochrane Database of Systematic Reviews, MEDLINE, ACP Journal Club, Database of Abstracts of Reviews, EMBASE, EBM Reviews, and Cochrane Library.

Search Strategy:

1     exp text messaging/ (849)

2     (text*messag* or texting*).mp. (746)

3     (texts adj10 cell).mp. (32)

4     (texts adj10 phone*).mp. (48)

5     (short messag* service or SMS message* or SMS).mp. (7288)

6     (messag* service or messag* delivery or text program*).mp. (893)

7     (extended messag* service or enhanced messag* service).mp. (0)

8     multimedia messag* service*.mp. (62)

9     message* text*.mp. (33)

10     (tailored adj10 messag*).mp. (851)

11     (sexting or sext).mp. (72)

12     or/1-11 (9206)

13     (or/1-9) or 11 (8427)

14     (cellular adj10 phone*).mp. (5394)

15     exp "cellular phone"/ (10778)

16     exp wireless technology/ (1925)

17     exp "computers,handheld"/ (16370)

18     iPhone*.mp. (498)

19     iPad*.mp. (817)

20     smartphone*.mp. (1079)

21     (android* or BlackBerry or BlackBerries or Google Nexus).mp. (2226)

22     ((portable adj5 phone*) or (transportable adj5 phone*)).mp. (97)

23     (Personal Digital Assistant* or microcomputer* or PC pocket* or (pilot adj5 palm*) or palm-top* or (palm adj5 pilot*) or (tablet adj5 PC)).mp. (35240)

24     IT communication device*.mp. (16)

25     (mobile adj10 technolog*).mp. (2345)

26     (mobile phone* or mobile cell*phone*).mp. (9774)

27     ((mobile adj5 application*) or (mobile adj5 technolog*)).mp. (2646)

28     ((digital adj5 device*) or (mobile adj5 device*)).mp. (4156)

29     (communication adj5 modalit*).mp. (448)

30     or/14-29 (58199)

31     exp telehealth/ (33673)

32     (telehealth or telemedic* or telemetry).mp. (50109)

33     (mobile adj5 health).mp. (4802)

34     or/31-33 (64451)

35     12 and (30 or 34) (1619)

36     13 and (30 or 34) (1569)

37     exp sexually transmitted disease/ or STD.mp. (369990)

38     sex*.mp. (1640167)

39     (sexually transmitted infection* or STI or sexually transmissible infection*).mp. (25384)

40     vener* disease*.mp. (9545)

41     exp acquired immunodeficiency syndrome/ (202178)

42     (HIV infection* or HIV seroprevalence or HIV-2 or seronegativity or HIV or HIV-1 or HIV-i or AIDS or seropositivity).mp. (707059)

43     (htlv adj10 infection*).mp. (9771)

44     ((CD4 or T-lymphocyte or T*cell) and (cell count or count)).mp. (94629)

45     viral [load.mp](http://load.mp). (54938)

46     exp anti-hiv agents/ (119689)

47     (anti-aids drug* or anti-hiv or highly active antiretroviral therapy or HAART or antiretroviral therap*).mp. (122898)

48     (ZIDOVUDINE or Reverse Transcriptase Inhibitor*).mp. (66143)

49     (3'-azido-2'3'-dideoxythymidine or bw a509u or bwa509u or azt antiviral or retrovir antiviral azt or bwa 509u or 3' azido 3' deoxythymidine).mp. or azt (antiviral) or [azidothymidine.mp](http://azidothymidine.mp). or 3'-azido-3'-[deoxythymidine.mp](http://deoxythymidine.mp). or 3' azido 2'3' [dideoxythymidine.mp](http://dideoxythymidine.mp). or [bwa-509u.mp](http://bwa-509u.mp). or [zidovudine.mp](http://zidovudine.mp). (48728)

50     (pre-exposure prophylaxis or PrEP).mp. (5305)

51     (Deoxycytidine or Organophosphonates).mp. (51934)

52     exp Gonorrhea/ or (Neisseria gonorrh* or clap or gonococcus).mp. (42057)

53     exp chlamydia/ or (chlamydia trachomatis or nongonococcal urethritis).mp. (37182)

54     exp Herpes Genitalis/ or genital herpes [simplex.mp](http://simplex.mp). (9843)

55     Trichomonas vaginali*.mp. or exp Trichomona*/ (10780)

56     exp SYPHILIS/ or (great pox or latent stage syphili* or TREPONEMA PALLIDUM).mp. (49610)

57     Neurosyphilis.mp. (6026)

58     exp Human Papilloma Virus/ or (HPV or Papillomavirus or Papillomaviridae or ALPHAPAPILLOMAVIRUS or BETAPAPILLOMAVIRUS or GAMMAPAPILLOMAVIRUS or MUPAPILLOMAVIRUS).mp. (81916)

59     exp Condylomata Acuminata/ or genital wart*.mp. or vener* wart*.mp. (13063)

60     exp pelvic infection/ or exp pelvic inflammatory disease/ or exp pelvic inflammatory disease/ or [adnexitis.mp](http://adnexitis.mp). (21843)

61     exp genital diseases,male/ or exp Genital Diseases, Female/ or exp Vaginosis, Bacterial/ or exp Vaginitis/ or exp Vaginitis/ or [vaginitides.mp](http://vaginitides.mp). or exp Vaginal Diseases/ or exp Uterine Cervicitis/ or exp Urethritis/ or exp Urinary Tract Infections/ (1451044)

62     exp reproductive health/ or sex* [health.mp](http://health.mp). or exp sex* behaviour*/ or oral [sex.mp](http://sex.mp). or anal [sex.mp](http://sex.mp). or vaginal [sex.mp](http://sex.mp). or sex* [intercourse.mp](http://intercourse.mp). (47607)

63     (sex* partner* or sex* activit* or condom*).mp. (76554)

64     (safe sex or unsafe sex or protected sex or sex education).mp. (26606)

65     (sex* worker* or prostitute*).mp. (11476)

66     exp homosexuality,male/ or exp homosexuality,female/ or bisex*.mp. or [lesbian.mp](http://lesbian.mp). or gay*.mp. or transgender*.mp. or [queer.mp](http://queer.mp). (34690)

67     "men who have sex with men".mp. [mp: ti, ab, tx, kw, ct, ot, sh, hw, tn, dm, mf, dv, ac, de, md, sd, so, nm, kf, ps, rs, ui] (9723)

68     "women who have sex with women".mp. [mp: ti, ab, tx, kw, ct, ot, sh, hw, tn, dm, mf, dv, ac, de, md, sd, so, nm, kf, ps, rs, ui] (209)

69     (risk* behaviour or coit* or risky partner* or hook* up or sex* intercourse).mp. (46365)

70     circumcision*.mp. (13545)

71     or/37-70 (3847791)

72     (vaginal smear* or pap smear* or papanicolau test*).mp. (31310)

73     (test* or screen* or smear* or serostatus* or diagnos* or urinal test* or blood test* or Anonymous test* or Outreach test*).mp. (10572531)

74     or/72-73 (10572533)

75     71 and 74 (1349690)

76     71 or 75 (3847791)

**77     12 and 76 (1221)**

78     30 and 76 (3650)

79     34 and 76 (3005)

80     35 and 76 (356)

81     78 and 79 (231)

**82     (12 or 30) and 76 (4529)**

**83     (12 or 30 or 34) and 76 (7289)**
