# Supplementary material for: Effectiveness of text messaging interventions on prevention, detection, treatment, and knowledge outcomes for sexually transmitted infections (STIs)/HIV: a systematic review and meta-analysis
Source: Syst Rev. 2019 Jan 8;8:12. doi: 10.1186/s13643-018-0921-4 (PMC6323863; doi:10.1186/s13643-018-0921-4)
Supplement: Supplementary file 3 — Ovid Search Strategy Phase 2. (DOCX 18 kb) [file 13643_2018_921_MOESM3_ESM.docx]

**Supplementary file 3: Ovid Search Strategy Phase 2**

Database: Ovid MEDLINE(R) 1946 to Present with Daily Update

Date Search Conducted: March 30, 2017

1. exp text messaging/ (1496)
2. (text*messag* or texting*).mp. (382)
3. (texts adj10 cell).mp. (19)
4. (texts adj10 phone*).mp. (36)
5. (short messag* service or SMS message* or SMS).mp. (3669)
6. (messag* service or messag* delivery or text program*).mp. (662)
7. (extended messag* service or enhanced messag* service).mp. (0)
8. multimedia messag* service*.mp. (32)
9. message* text*.mp. (15)
10. (tailored adj10 messag*).mp. (511)
11. (sexting or sext).mp. (70)
12. or/1-11 (5486)
13. (or/1-9) or 11 (5057)
14. (cellular adj10 phone*).mp. (698)
15. exp "cellular phone"/ (8737)
16. exp wireless technology/ (2321)
17. exp "computers,handheld"/ (3882)
18. iPhone*.mp. (389)
19. iPad*.mp. (684)
20. smartphone*.mp. (2720)
21. (android* or BlackBerry or BlackBerries or Google Nexus).mp. (1485)
22. ((portable adj5 phone*) or (transportable adj5 phone*)).mp. (55)
23. (Personal Digital Assistant* or microcomputer* or PC pocket* or (pilot adj5 palm*) or palm-top* or (palm adj5 pilot*) or (tablet adj5 PC)).mp. (17288)
24. IT communication device*.mp. (0)
25. (mobile adj10 technolog*).mp. (1906)
26. (mobile phone* or mobile cell*phone*).mp. (4014)
27. ((mobile adj5 application*) or (mobile adj5 technolog*)).mp. (3869)
28. ((digital adj5 device*) or (mobile adj5 device*)).mp. (2783)
29. (communication adj5 modalit*).mp. (251)
30. or/14-29 (36817)
31. exp telehealth/ (21252)
32. (telehealth or telemedic* or telemetry).mp. (29397)
33. (mobile adj5 health).mp. (5013)
34. or/31-33 (37674)
35. 30 or 34 (70681)
36. 12 and 35 (1988)
37. 13 and 35 (1954)
38. exp sexually transmitted disease/ or STD.mp. (315039)
39. sex*.mp. (852220)
40. (sexually transmitted infection* or STI or sexually transmissible infection*).mp. (12427)
41. vener* disease*.mp. (4970)
42. exp acquired immunodeficiency syndrome/ (75889)
43. (HIV infection* or HIV seroprevalence or HIV-2 or seronegativity or HIV or HIV-1 or HIV-i or AIDS or seropositivity).mp. (376597)
44. (htlv adj10 infection*).mp. (5742)
45. ((CD4 or T-lymphocyte or T*cell) and (cell count or count)).mp. (39394)
46. viral [load.mp](http://load.mp). (35903)
47. exp anti-hiv agents/ (60531)
48. (anti-aids drug* or anti-hiv or highly active antiretroviral therapy or HAART or antiretroviral therap*).mp. (70402)
49. (ZIDOVUDINE or Reverse Transcriptase Inhibitor*).mp. (24015)
50. (3'-azido-2'3'-dideoxythymidine or bw a509u or bwa509u or azt antiviral or retrovir antiviral azt or bwa 509u or 3' azido 3' deoxythymidine).mp. or azt (antiviral) or [azidothymidine.mp](http://azidothymidine.mp). or 3'-azido-3'-[deoxythymidine.mp](http://deoxythymidine.mp). or 3' azido 2'3' [dideoxythymidine.mp](http://dideoxythymidine.mp). or [bwa-509u.mp](http://bwa-509u.mp). or [zidovudine.mp](http://zidovudine.mp). (12352)
51. (pre-exposure prophylaxis or PrEP).mp. (3005)
52. (Deoxycytidine or Organophosphonates).mp. (29430)
53. exp Gonorrhea/ or (Neisseria gonorrh* or clap or gonococcus).mp. (20041)
54. exp chlamydia/ or (chlamydia trachomatis or nongonococcal urethritis).mp. (16606)
55. exp Herpes Genitalis/ or genital herpes [simplex.mp](http://simplex.mp). (4651)
56. Trichomonas vaginali*.mp. or exp Trichomona*/ (4632)
57. exp SYPHILIS/ or (great pox or latent stage syphili* or TREPONEMA PALLIDUM).mp. (28230)
58. Neurosyphilis.mp. (3357)
59. exp Human Papilloma Virus/ or (HPV or Papillomavirus or Papillomaviridae or ALPHAPAPILLOMAVIRUS or BETAPAPILLOMAVIRUS or GAMMAPAPILLOMAVIRUS or MUPAPILLOMAVIRUS).mp. (43590)
60. exp Condylomata Acuminata/ or genital wart*.mp. or vener* wart*.mp. (6085)
61. exp pelvic infection/ or exp pelvic inflammatory disease/ or exp pelvic inflammatory disease/ or [adnexitis.mp](http://adnexitis.mp). (10837)
62. exp genital diseases,male/ or exp Genital Diseases, Female/ or exp Vaginosis, Bacterial/ or exp Vaginitis/ or exp Vaginitis/ or [vaginitides.mp](http://vaginitides.mp). or exp Vaginal Diseases/ or exp Uterine Cervicitis/ or exp Urethritis/ or exp Urinary Tract Infections/ (681345)
63. exp reproductive health/ or sex* [health.mp](http://health.mp). or exp sex* behaviour*/ or oral [sex.mp](http://sex.mp). or anal [sex.mp](http://sex.mp). or vaginal [sex.mp](http://sex.mp). or sex* [intercourse.mp](http://intercourse.mp). (17037)
64. (sex* partner* or sex* activit* or condom*).mp. (42882)
65. (safe sex or unsafe sex or protected sex or sex education).mp. (18098)
66. (sex* worker* or prostitute*).mp. (6413)
67. exp homosexuality,male/ or exp homosexuality,female/ or bisex*.mp. or [lesbian.mp](http://lesbian.mp). or gay*.mp. or transgender*.mp. or [queer.mp](http://queer.mp). (21664)
68. "men who have sex with men".mp. [mp: ti, ab, tx, kw, ct, ot, sh, hw, tn, dm, mf, dv, ac, de, md, sd, so, nm, kf, ps, rs, ui] (6333)
69. "women who have sex with women".mp. [mp: ti, ab, tx, kw, ct, ot, sh, hw, tn, dm, mf, dv, ac, de, md, sd, so, nm, kf, ps, rs, ui] (127)
70. (risk* behaviour or coit* or risky partner* or hook* up or sex* intercourse).mp. (19553)
71. circumcision*.mp. (6894)
72. or/37-70 (1902462)
73. (vaginal smear* or pap smear* or papanicolau test*).mp. (24156)
74. (test* or screen* or smear* or serostatus* or diagnos* or urinal test* or blood test* or Anonymous test* or Outreach test*).mp. (5221675)
75. or/73-74 (5221675)
76. 72 and 75 (650616)
77. 72 or 77 (1902462)
78. **12 and 76 (825)**
79. 30 and 77 (2615)
80. 34 and 77 (1592)
81. 35 and 77 (3975)
82. 79 and 80 (232)
83. 12 or 30 (40372)
84. 12 or 30 or 34 (72179)
85. 77 and 83 (3028)
86. **77 and 84 (4382)**
87. (201308$ or 201310$ or 201311$ or 201312$).ed. (305526)
88. 86 and 87 (114)
89. Limit 86 to yr=”2014-Current” (1167)
90. **88 or 89 (1281)**

**Database:** <https://www.mhealthevidence.org>

**Date Search Conducted:** March 30, 2017

Due to the limited search functions of the website we were only able to conduct a basic search: (sms or text or texting) and random
