# Supplementary material for: Effectiveness of text messaging interventions on prevention, detection, treatment, and knowledge outcomes for sexually transmitted infections (STIs)/HIV: a systematic review and meta-analysis
Source: Syst Rev. 2019 Jan 8;8:12. doi: 10.1186/s13643-018-0921-4 (PMC6323863; doi:10.1186/s13643-018-0921-4)
Supplement: Supplementary file 4 — Eligibility Criteria. (DOCX 14 kb) [file 13643_2018_921_MOESM4_ESM.docx]

**Supplementary file 4: Eligibility Criteria**

*Eligibility criteria*

All RCTs examining the effect of SMS on STI/HIV outcomes among adults and youth published in English between January 1, 1996 and March 31, 2017 were included. We included only RCTs published in English as we did not have the financial and human resources to translate non-English studies. We included all outcomes related to the prevention and treatment of STI/HIV.

Our PICO (Population, Intervention, Comparison, Outcome) inclusion criteria were:

- (P) adults and youth who were at risk of acquiring (or who currently have) a STI and/or HIV,
- (I) text messages (one-way or two-way) sent by a health care provider aimed at improving sexual health outcomes,
- (C) standard of care, no text messages, or alternate intervention with equivalent aims, and
- (O) adherence to STI/HIV treatments and/or vaccination, measures of behaviour change (such as condom use), uptake of STI/HIV testing, knowledge of sexual health and HIV medication, appointment adherence.

We included only RCTs published in English as we did not have the financial and human resources to translate non-English studies. We excluded non-randomised studies and publications before 1996. **Studies were excluded if they were not primary studies (such as review articles, conference abstract and posters, editorials).**
