# Supplementary material for: Effectiveness of text messaging interventions on prevention, detection, treatment, and knowledge outcomes for sexually transmitted infections (STIs)/HIV: a systematic review and meta-analysis
Source: Syst Rev. 2019 Jan 8;8:12. doi: 10.1186/s13643-018-0921-4 (PMC6323863; doi:10.1186/s13643-018-0921-4)
Supplement: Supplementary file 5 — Table of SMS Messages. (DOCX 21 kb) [file 13643_2018_921_MOESM5_ESM.docx]

**Supplementary file 5: Content of SMS messages**

| **Study** | **Content of messages** |
| --- | --- |
| Da Costa 2012 | The UNIFESP informs: take good care of your health.[reminder to take meds] |
| Davey 2016 | With health there is joy. Love yourself. Come to your Health Center for your next visit scheduled for xx-xx-xxxx day! |
| De Tolly 2012 | Informational-style SMS: “In SA 1,400 people get HIV every day. Test for HIV so you know if you’re one (so then u can look after yourself) or if you’re negative make sure u stay that way.”  Motivational-style SMS: “If you test and you’re HIV+ you can go on free drugs when you need to. HIV is no longer a death sentence. You can live a long, normal life with HIV. Plz test!”  Request for participants to indicate whether they tested: “Thanks for being part of our research study. We don’t need to know your HIV status! We just want to know if u tested or not. U will earn airtime. If u HAVE tested for HIV after 22 September this year send a please-call-me to 0714331022. Plz send this before 21 November. If u have NOT tested for HIV after 22 September this year send a plz-call-me to 0714549042. Plz send this before 21 November. If u do this u will earn R10 airtime (whether u tested or not!!). We will load it in a week or two. Questions? Call 0214691111” |
| Garofalo 2016 | Message delivery was timed to coincide with individual dosing schedule. Examples of these messages designed by participants include: ‘‘Mission accomplished?’’; ‘‘Will you choose life?’’ A set of motivational or encouraging follow-up messages were randomized depending on their afﬁrmative or negative response (e.g., ‘‘Well done,’’ ‘‘You can do it!’’), respectively. This 2-way personalized message system was designed as youth-friendly, including the positive feedback loop to promote self-management, extending beyond the scope of many commercial products that send 1-way ‘‘canned’’ reminders. |
| Hardy 2011 | Topics were drawn from our ﬁndings from Phase 1, and feed sources were selected from the same categories and links published by leading web content providers, Yahoo and MSN. For example, The Onion provided a ‘‘jokes’’ feed, Accuweather provided a ‘‘weather’’ feed, and so forth. Consequently, content of re- minders could include popular news, sports, weather information, and other timely information. Participants chose one of the following categories at the time of their enrollment: news, weather, celebrity news, humor, jokes, Bible verses, word of the day, baseball, basketball, or football. They could select a different category during the study visits at week 3 and 6, although only 2 chose to do so. Upon receipt of the reminder message, the participant was instructed to respond with an SMS in order to conﬁrm that they were taking their |
| Ignersoll 2015 | Medication adherence queries said: “Did you take your medication today? Text back MEDS Y or MEDS N.” Mood queries said: “How is your mood right now? (0  extremely negative,1 = somewhat negative,2 = neutral,3 = somewhat positive,4 = extremely positive). Reply MOOD #.” Privacy of substance use messages sent back and forth was a concern. Al- though phones could be password-protected if the user selected a password, messages could show on the screen even when the phone was locked. To minimize potential privacy concerns, we masked substance use queries by using a weather question. Spe- cifically, participants were asked “How were the skies in the past 24 hours? Respond SKIES clear, cloudy, rainy, snowy, or other.” Participants responded SKIES clear if they were reporting no drinking or drug use in the past 24 hours, cloudy if they smoked marijuana, rainy if they drank alcohol, snowy if they used crack or cocaine, and other if they used other illicit drugs. They could also report use of multiple substances to the SKIES query (i.e., “rainy and cloudy”). |
| Lester 2010 | On Monday morning of each week, the site nurse or clinical oﬃ cer sent a text message via SMS to patients in the intervention group to inquire about their status and thus to remind them about the availability of phone-based support. Typically, the slogan “Mambo?” was sent, which is Kiswahili for “How are you?” The health workers used multiple recipient (bulk) messaging functions to improve eﬃ ciency. Patients in the inter- vention group were instructed to respond within 48 h that either they were doing well (“Sawa”) or that they had a problem (“Shida”). The clinician then called patients who said they had a problem or who failed to respond within 2 days. Participants were instructed that health- care workers were available to respond during clinic hours only. All mobile phone communications between the health-care workers and patients were recorded in the study log. |
| Lim 2010 | The SMS were short and catchy pieces of advice or information about STI or safe sex - for example, ‘Chlamydia: hard to spell, easy to catch -Use a condom’. |
| Mbuagbaw 2012 | The content was varied and contemporary (e.g. messages would contain season’s greetings) so as to retain participants’ attention throughout the study period and to explore the various aspects of behavior change. An example of a message would be, ‘‘You are important to your family. Please remember to take your medication. You can call us at this number: +237 xxxx xxxx.’’ The messages made no mention of HIV. We used a series of 11 messages that were changed every week. |
| Moore 2015 | Stay healthy! It’s time 2 take ur meds, pls take ur [med] now.  It’s pill time! Take ur [med] now.  Taking ur meds helps control ur disease. Rmber 2 take ur [med] now.  It’s med time, only u can control this. Rmber 2 take ur [med] now.  Adherence is impt. Pls take ur… |
| Morris 2015 | The content of the e-mail and text messages included the participants’ names, a reminder to vaccinate, and steps on howto report becoming UTD or to unsubscribe from the reminder ser- vice. The reminders were sent in either English or Spanish depending on the recipient’s preference. None of the reminders speciﬁed which vaccines were needed. |
| Mugo 2016 | The first SMS read: “Please remember to go for your clinic appointment tomorrow. Call this number if you need more information”, while the second SMS read: “You missed your clinic appointment yesterday. Please report to the clinic as soon as possible.” |
| Norton 2014 | Messages were sent 1 day prior to the appointment date and read ‘‘Remember: you have a doctor’s appointment tomorrow.’’ |
| Odeny 2012 | If u r not the intended recipient of this Male Circumcision (MC) message, please text STOP to 0722819835 and you will not receive future messages. Thank you.  This is your MC provider. It is normal to feel a bit of pain and swelling, but if there is severe swelling, bleeding or pain please come back to the clinic.  This is your MC provider. Remember do not allow water to soak the dressing before removal on the 3rd day. Remove the dressing today. Make sure you review the post-op instructions and use the blade provided. Throw away the blade after use.  This is your MC provider. Always keep the genital area dry n clean to avoid infection. Do not apply any ointment or creams that are not prescribed by the clinic  This is your MC provider. If you feel heavy pain, swelling, bleeding, or any signs of infection please consult the clinic.  This is your MC provider. Don’t forget to come back to the clinic for your day 7 follow-up visit. You will be checked to be certain the healing is going well.  This is your MC provider. See you at the clinic today for your follow-up visit. |
| Orrell 2015 | The ﬁrst 5 participants randomized were asked to construct a simple message that would remind them to take their tablets, but not disclose their HIV status to others at home or in the community. These message options were then made available for the rest of the cohort, in either English or Xhosa. Once each participant in the intervention arm had chosen a message, they received the same message through- out the study when their dosing was late. Messages included “Have you forgotten something?” (Awulibelanga nto?), “Just take it!” (Yithathe!), “Wake up” (Vuka!), It’s8 o’clock! (Ngu 8o’clock!), or their study number (eg, XX9999). |
| Patel 2014 | All messages reminded patients to schedule their next vaccine and provided health center con- tact information. For example, the text message for participants at Planned Parenthood sites stated: “Reminder: schedule your next HPV Vaccine if you have not done so. Call 1-800-230-PLAN or go to www.plannedparenthood.org. You will get a total of 4 reminders.” |
| Pop-Eleches 2011 | Short reminder: This is your reminder.  Long reminder: This is your reminder. Be strong and courageous, we care about you. |
| Rand 2015 | An initial notice informed parents that theywere enrolled in a healthmessage programfromtheir child’s insurer: “MPHealth: Ur opted in 4 health messages frm ur child’s insurance [name of insurance plan] THX! Msg&Data Rates May Apply Text STOP to opt-out.” If participants did not opt out, a reminder for anHPV vaccination (MPHealth:‘Your [age] yr old is due for an HPV vaccine. Pls call [phone number and ofﬁce name] to schedule an appt. Text STOP to opt-out)’ was sent. |
| Rand 2017 | The phone message stated “Hello, this is a reminder message that your xx-year old is due for their next HPV vaccination. Please call xxx-xxxx to schedule your appointment. If you have already done so, please disregard this message.” The text message was shortened to “Reminder your xx-year-old child is due for their next HPV vaccine. Call xxx-xxxx to schedule. If you have already done so, please ignore this message.” Reminders continued if a patient had not yet completed the vaccine series through April 2014. |
| Sabin 2015 | The text messages were personalized, with subjects selecting from a list of 10 options developed jointly by clinicians and patients, including “carry on, carry on!” and “be healthy, have a happy family.” To prevent disclosure of HIV, text reminders did not refer to HIV, ART, or other disease-related topics. |
| Shet 2014 | The main aspect of the intervention was a customised motivational voice call that went out once a week at a time selected by each patient. The patient also chose the sex and language of the pre-recorded voice call. This automated call began with a greeting and the hope that the patient was feeling well, followed by an inquiry whether medications were taken as prescribed. The message was considered interactive or bidirectional, since it required the patient to respond to a question about the previous day’s pill doses, by pressing “1” for yes or “2” for no. If the patient failed to respond to the call, a maximum of three more calls were made over the ensuing 24 hours until a response was obtained. The second aspect of the intervention included a weekly non-interactive neutral pictorial message sent out as a reminder four days after the automated call. |
