# Supplementary material for: Effectiveness of text messaging interventions on prevention, detection, treatment, and knowledge outcomes for sexually transmitted infections (STIs)/HIV: a systematic review and meta-analysis
Source: Syst Rev. 2019 Jan 8;8:12. doi: 10.1186/s13643-018-0921-4 (PMC6323863; doi:10.1186/s13643-018-0921-4)

**Supplementary file 6: Risk of Bias Figures**

Risk of bias items presented as percentages across all included studies


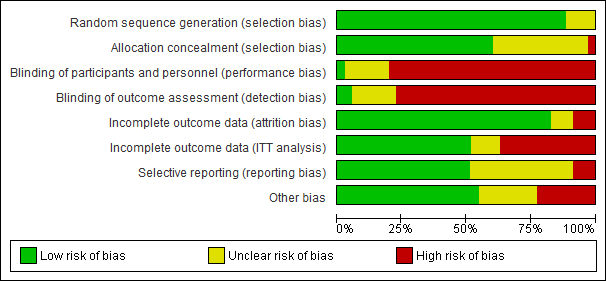


Figure 3: Risk of bias items for each included study


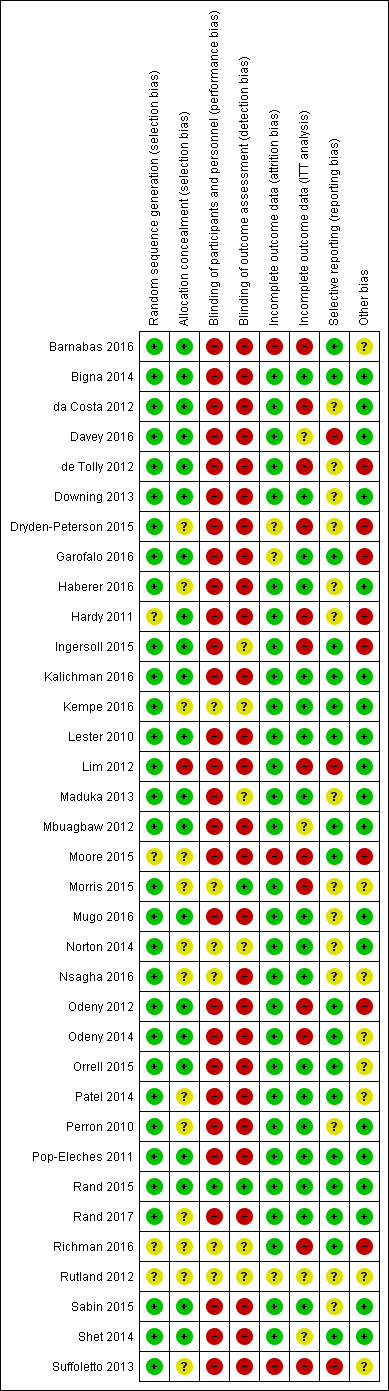

Supplement: Supplementary file 6 — Risk of Bias Figures. (DOCX 47 kb) [file 13643_2018_921_MOESM6_ESM.docx]
