# Supplementary material for: Effectiveness of text messaging interventions on prevention, detection, treatment, and knowledge outcomes for sexually transmitted infections (STIs)/HIV: a systematic review and meta-analysis
Source: Syst Rev. 2019 Jan 8;8:12. doi: 10.1186/s13643-018-0921-4 (PMC6323863; doi:10.1186/s13643-018-0921-4)
Supplement: Supplementary file 7 — Publication Bias Analysis and Subgroup Analysis Table. (DOCX 26 kb) [file 13643_2018_921_MOESM7_ESM.docx]

**Supplementary file 7: Publication Bias Analysis and Subgroup Analysis table**

**1. Publication Bias Analysis**

Medication adherence

. metabias var2 var3 var4 var5, egger

Warning: varlist has 3 variables but option 'ci' not specified; 'ci' assumed.

Tests for Publication Bias

Begg's Test

adj. Kendall's Score (P-Q) = -3

Std. Dev. of Score = 20.21

Number of Studies = 15

z = -0.15

Pr > |z| = 0.882

z = 0.10 (continuity corrected)

Pr > |z| = 0.921 (continuity corrected)

Egger's test

------------------------------------------------------------------------------

Std_Eff | Coef. Std. Err. t P>|t| [95% Conf. Interval]

-------------+----------------------------------------------------------------

slope | 4.409557 .0655994 67.22 0.000 4.267838 4.551276

bias | 1.529112 .4067533 3.76 0.002 .6503752 2.407849

------------------------------------------------------------------------------

**3. Subgroup Analysis table**

| **Outcomes** | **Low risk** | **High risk** | **Population** |
| --- | --- | --- | --- |
| Appointment adherence | Bigna 2014 |  | Adults |
| Appointment adherence |  | Davey 2016 | Adults |
| Appointment adherence |  | Ignersoll 2015 | Adults |
| Appointment adherence |  | Mugo 2016 | 18–29 year old patients |
| Appointment adherence |  | Norton 2014 | Adults |
| Appointment adherence |  | Odeny 2012 | Adults |
| Appointment adherence |  | Perron 2010 | Adults |
| Appointment adherence |  | Rutland 2012 | aged 16–30 years |
| ART initiation before 30 weeks gestation |  | Dryden-Peterson 2015 | Adults |
| CD4 cell count increase |  | Moore 2015 | Adults |
| Completion of HPV Vaccine series |  | Kempe 2016 | Adults |
| Completion of HPV Vaccine series |  | Morris 2015 | Adults |
| Completion of HPV Vaccine series |  | Patel 2014 | Females 19-26 |
| Completion of HPV Vaccine series |  | Rand 2017 | Adults |
| Completion of HPV Vaccine series |  | Richman 2016 | Uni students ages of 18 and 26 |
| Completion of HPV Vaccine series | Rand 2015 |  | Adults |
| Condom use |  | Lim 2012 | 16-29 year old patients |
| Condom use |  | Suffoleto 2013 | Female patients aged 18-25 years |
| Education of sexual health |  | Downing 2013 | Chlamydia+ adults |
| Education of sexual health |  | Odeny 2014 | Adults |
| HIV adherence - pill count |  | Da Costa 2012 | HIV-positive Brazilian women |
| HIV adherence - pill count |  | Haberer 2016 | Adults |
| HIV adherence - pill count |  | Hardy 2011 | Adults |
| HIV adherence - pill count |  | Ignersoll 2015 | Adults |
| HIV adherence - pill count |  | Moore 2015 | Adults |
| HIV adherence - pill count |  | Sabin 2015 | Adults |
| HIV adherence - pill count |  | Shet 2014 | Adults |
| HIV adherence - pill count | Kalichman 2016 |  | Adults |
| HIV adherence - pill count | Orrell 2015 |  | Adults |
| HIV adherence - pill count | Pop-Eleches 2011 |  | Adults |
| HIV adherence - self eported |  | Garofalo 2016 | Adults |
| HIV adherence - self reported |  | Maduka 2013 | Adults |
| HIV adherence - self reported |  | Mbuaghaw 2012 | Adults |
| HIV adherence - self reported |  | Nsagha 2016 | Adults |
| HIV adherence - self reported | Lester 2010 |  | Adults |
| Knowledge of sexual health |  | Lim 2012 | Adults |
| Presence of a new opportunistic infection |  | Mbuaghaw 2012 | Adults |
| Uptake of CD4 testing |  | Dryden-Peterson 2015 | Adults |
| Uptake of circumcision |  | Barnabas 2016 | Men 16-49 years |
| Uptake of HIV testing |  | de Tolly 2012 | Adults |
| Uptake of HIV testing |  | Mugo 2016 | Adults |
| Viral load suppression |  | Moore 2015 | Adults |
| Viral load suppression |  | Sabin 2015 | Adults |
| Viral load suppression |  | Shet 2014 | Adults |
| Viral load suppression | Lester 2010 |  | Adults |
| Viral load suppression | Orrell 2015 |  | Adults |
