# Supplementary material for: Effectiveness of text messaging interventions on prevention, detection, treatment, and knowledge outcomes for sexually transmitted infections (STIs)/HIV: a systematic review and meta-analysis
Source: Syst Rev. 2019 Jan 8;8:12. doi: 10.1186/s13643-018-0921-4 (PMC6323863; doi:10.1186/s13643-018-0921-4)
Supplement: Supplementary file 8 — GRADE Assessment of other Outcomes. (DOCX 18 kb) [file 13643_2018_921_MOESM8_ESM.docx]

# Supplementary file 8: GRADE assessment of STI and HIV outcomes

| **Certainty assessment** | | | | | | | **№ of patients** | | **Effect** | | **Certainty** | **Importance** |
| --- | --- | --- | --- | --- | --- | --- | --- | --- | --- | --- | --- | --- |
| **№ of studies** | **Study design** | **Risk of bias** | **Inconsistency** | **Indirectness** | **Imprecision** | **Other considerations** | **text messaging** | **control** | **Relative (95% CI)** | **Absolute (95% CI)** |  |  |
| Condom use | | | | | | | | | | | | |
| 2 | randomised trials | serious d | not serious | not serious | serious e |  | 28/232 (12.1%) | 41/263 (15.6%) | **OR 0.79**  (0.42 to 1.49) | **29 fewer per 1,000**  (from 60 more to 84  fewer) | - | IMPORTANT |
| Completion of HPV vaccination series (3 doses) | | | | | | | | | | | | |
| 6 | randomised trials | serious d | serious b | not serious | serious e |  | 447/2157 (20.7%) | 715/5207 (13.7%) | **OR 1.68**  (1.00 to 2.81) | **74 more per 1,000**  (from 0 fewer to 172  more) | - | IMPORTANT |
| Knowledge of S TIs | | | | | | | | | | | | |
| 1 | randomised trials | serious a | not serious | not serious | very serious e | publication bias strongly suspected | 95/217 (43.8%) | 92/242 (38.0%) | **OR 1.27**  (0.87 to 1.84) | **58 more per 1,000**  (from 32 fewer to 150  more) | ⨁◯◯◯  VERY LOW | IMPORTANT |
| Knowledge of ART dosage and dosing instructions | | | | | | | | | | | | |
| 1 | randomised trials | not serious | not serious | not serious | serious e |  | 14/52 (26.9%) | 11/52 (21.2%) | **OR 1.37**  (0.56 to 3.39) | **57 more per 1,000**  (from 81 fewer to 265  more) | - | IMPORTANT |
| Uptake of S TI/HIV testing | | | | | | | | | | | | |
| 6 | randomised trials | not serious | not serious | serious c | serious e |  | 470/998 (47.1%) | 372/1005 (37.0%) | **OR 1.73**  (1.39 to 2.15) | **134**  **more per 1,000**  (from 79 more to 188  more) | - | IMPORTANT |
| Uptake of circumcision | | | | | | | | | | | | |

| 1 | randomised trials | serious d | not serious | not serious | not serious |  | 137/288 (47.6%) | 168/462 (36.4%) | **OR 1.59**  (1.18 to 2.14) | **112**  **more per 1,000**  (from 39 more to 186  more) | - | IMPORTANT |
| --- | --- | --- | --- | --- | --- | --- | --- | --- | --- | --- | --- | --- |
| Presence of a new opportunistic infection | | | | | | | | | | | | |
| 1 | randomised trials | not serious | not serious | not serious | serious e |  | 36/101 (35.6%) | 26/99 (26.3%) | **OR 1.56**  (0.85 to 2.85) | **95 more per 1,000**  (from 30 fewer to 241  more) | - | IMPORTANT |
| Uptake of CD4 testing | | | | | | | | | | | | |
| 1 | randomised trials | serious d | not serious | not serious | serious e |  | 100/169 (59.2%) | 79/156 (50.6%) | **OR 1.41**  (0.91 to 2.19) | **85 more per 1,000**  (from 24 fewer to 186  more) | - | IMPORTANT |
| Viral load suppression | | | | | | | | | | | | |
| 5 | randomised trials | not serious | not serious | serious c | serious e |  | 572/796 (71.9%) | 545/780 (69.9%) | **OR 1.08**  (0.81 to 1.44) | **16 more per 1,000**  (from 46 fewer to 71  more) | - | IMPORTANT |
| ART initiation before 30 weeks gestation | | | | | | | | | | | | |
| 1 | randomised trials | serious d | not serious | not serious | serious e |  | 56/169 (33.1%) | 37/156 (23.7%) | **OR 1.59**  (0.98 to 2.60) | **94 more per 1,000**  (from 4 fewer to 210  more) | - | IMPORTANT |
| CD4 cell count increase | | | | | | | | | | | | |
| 2 | randomised trials | serious d | not serious | not serious | serious e |  | 56/93 (60.2%) | 48/84 (57.1%) | **OR 1.13**  (0.62 to 2.06) | **30 more per 1,000**  (from 119  fewer to 162  more) | - | IMPORTANT |

**CI:** Confidence interval; **OR:** Odds ratio

**Explanations**

1. S erious risk of bias. We downgraded because 7 out of ten RCTs were at high risk of bias.
2. S erious Inconsistency. There was unexplained inconsistency, moderate I2 values with statistically significant heterogeneity of effect estimates. One study's confidence intervals did not overlap (S abin 2015).
3. S erious indirectness. Populations studied varied, as well as frequency of text reminders sent.
4. S erious risk of bias. S tudies were at high risk of bias for the majority of domains.
5. S erious Imprecision. Results are imprecise as there is a wide confidence interval (CI) around the estimate of the effect
